# Supplementary material for: Cholinesterases Inhibition, Anticancer and Antioxidant Activity of Novel Benzoxazole and Naphthoxazole Analogs
Source: Molecules. 2022 Dec 3;27(23):8511. doi: 10.3390/molecules27238511 (PMC9738531; doi:10.3390/molecules27238511)

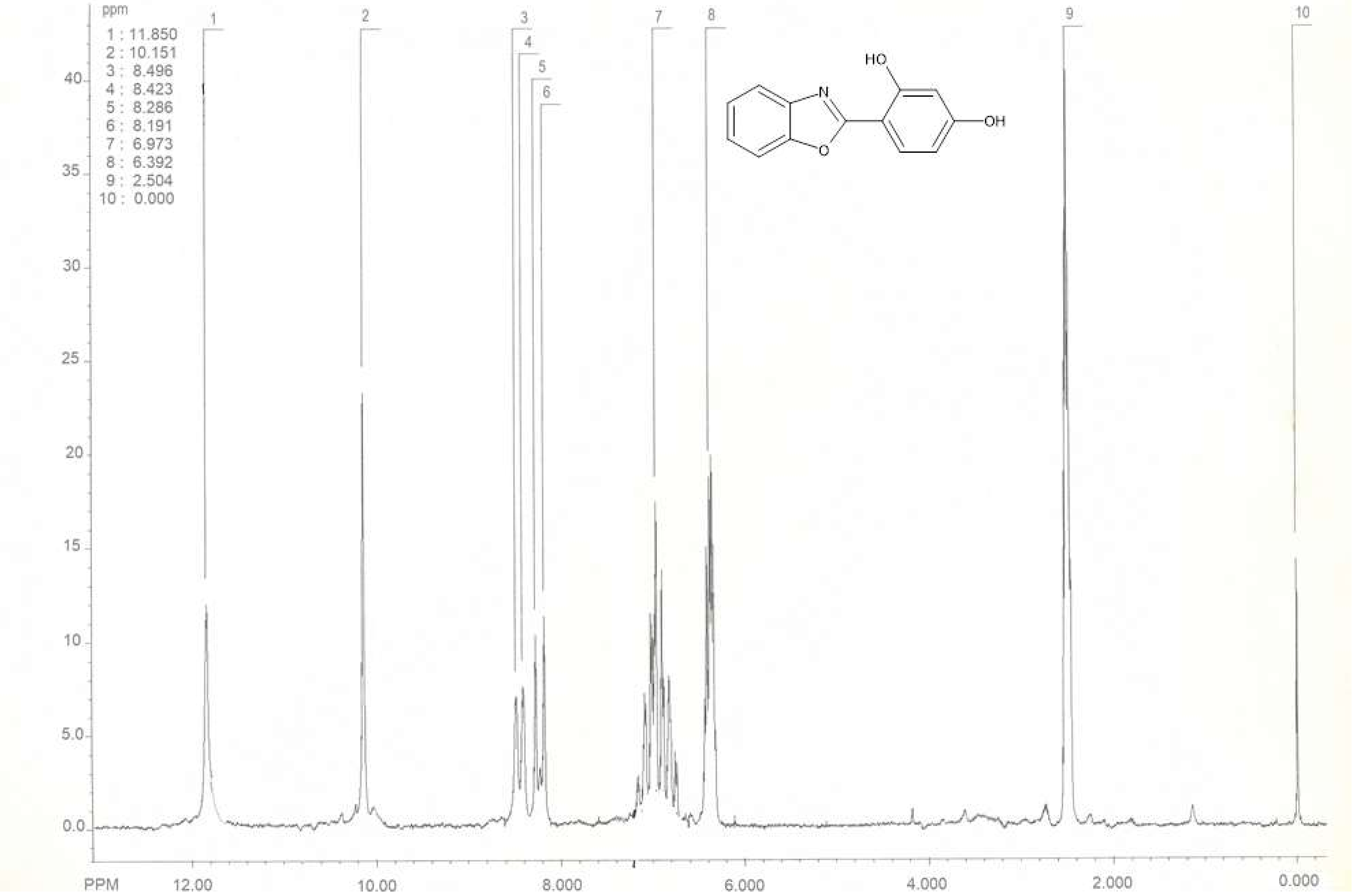
ppm
1 : 11.850
2 : 10.151
3 : 8.496
4 : 8.423
5 : 8.286
6 : 8.191
7 : 6.973
8 : 6.392
9 : 2.504
10 : 0.000
HO
N
O
OH
PPM
12.00
10.00
8.000
6.000
4.000
2.000
0.000

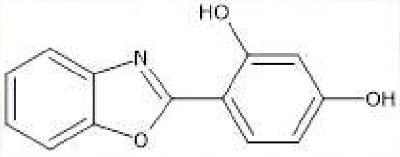

Spektrometr mas AMD-604

10:42 h

File : LRAN1 , Vers. 1

Comment : (EI 70eV 8kV 33-600 5s 5kHz) Nr 1

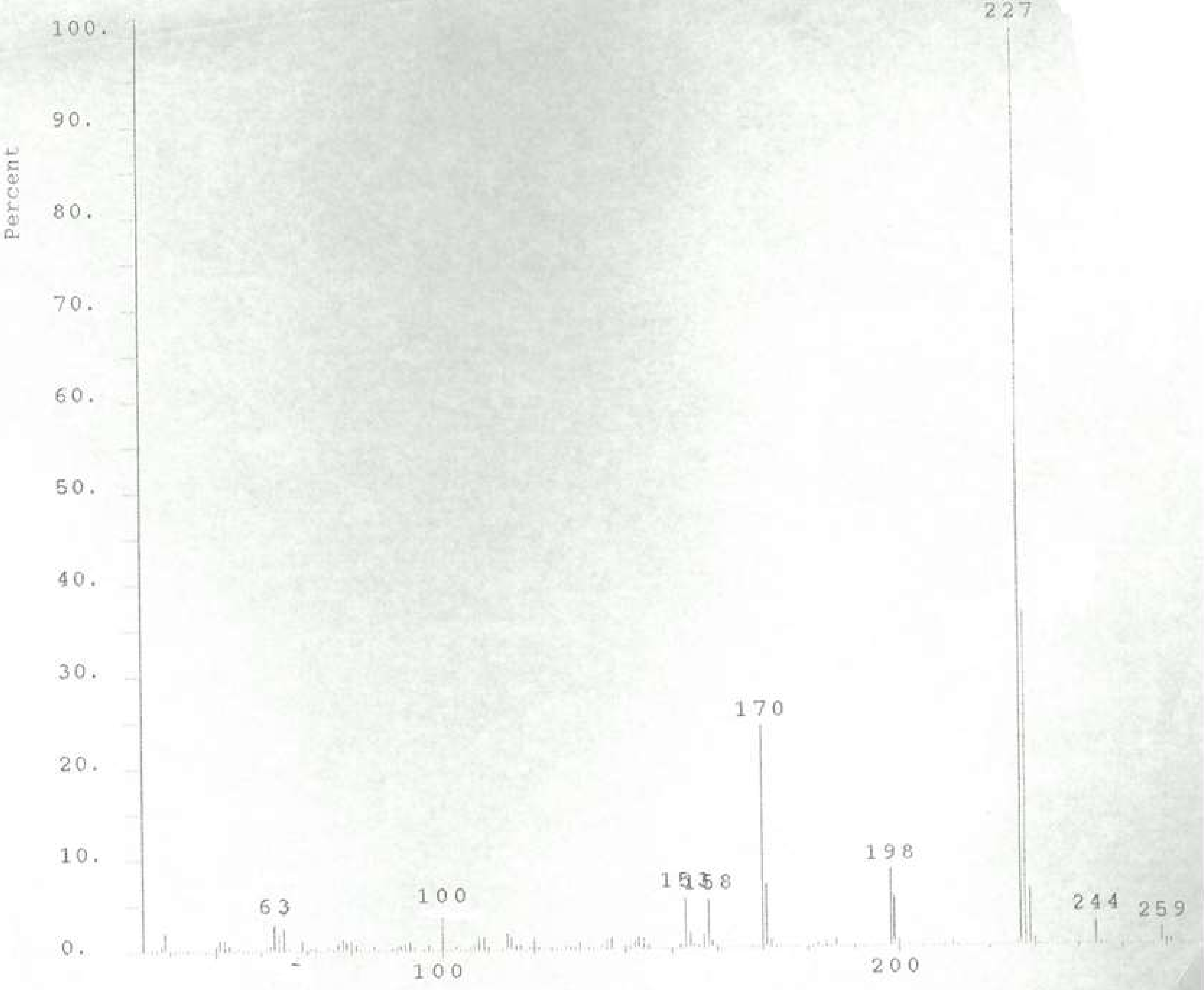

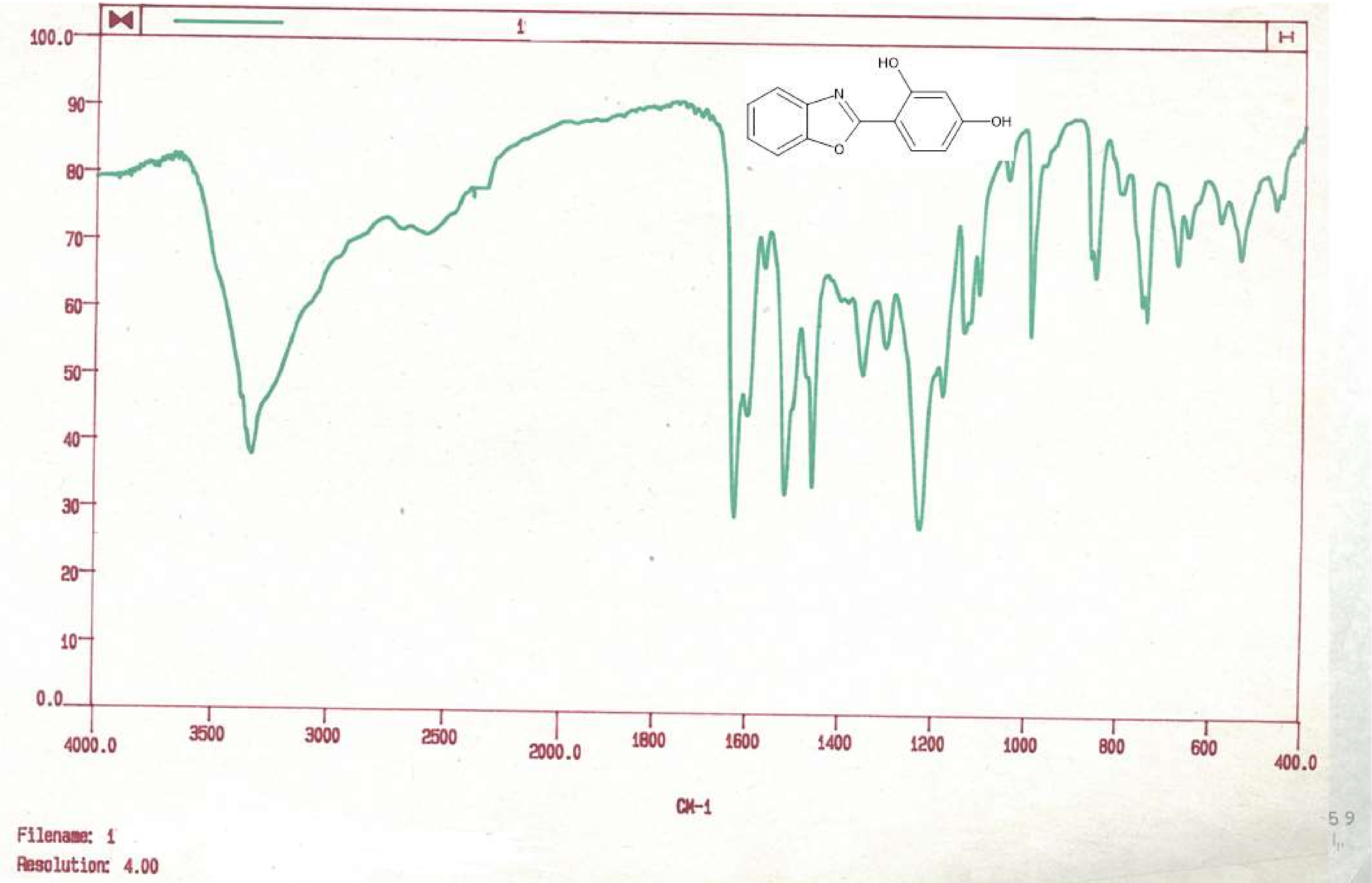

1
100.0
90
80
70
60
50
40
30
20
10
0.0
4000.0
3500
3000
2500
2000.0
1800
1600
1400
1200
1000
800
600
400.0
CM-1
HO
N
O
OH
Filename: 1
Resolution: 4.00

File Name : E:\Inne\LR_Lubl\an4481.ms2

File Type : Lo-Res Data - Ctd (Magnet)
File Source : Acquired on MASPEC II system [II32/99D9]
File Title : (EI 70 eV 33-800)
Operator : Małgorzata
Instrument : AMD 604

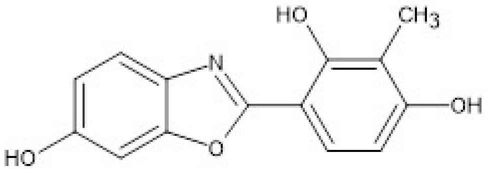

SCAN GRAPH. Flagging=Nominal M/z.
Scan 33#4:51. Entries=1010. Base M/z=257.1. 100% Int.=53.3248. Temp =222.

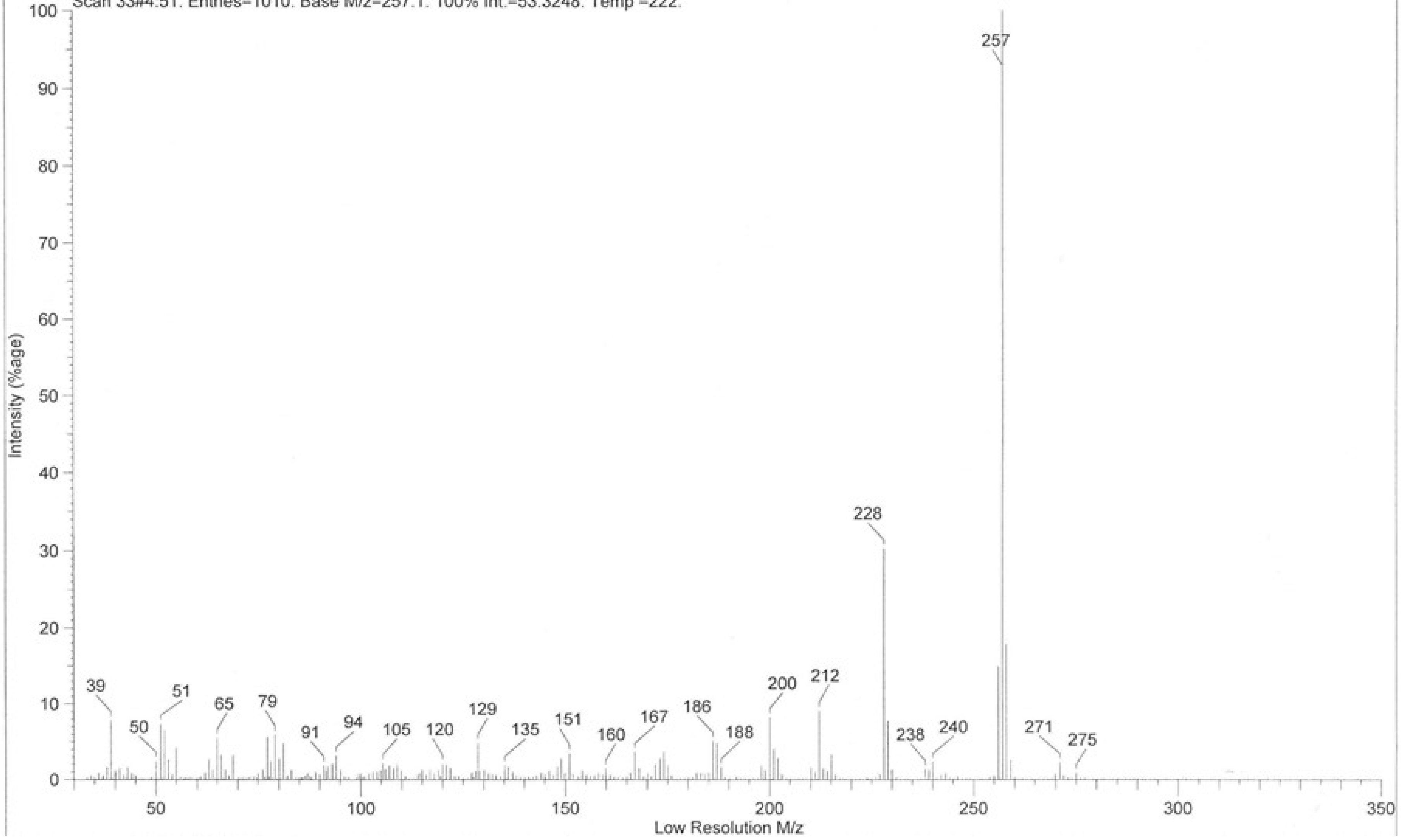

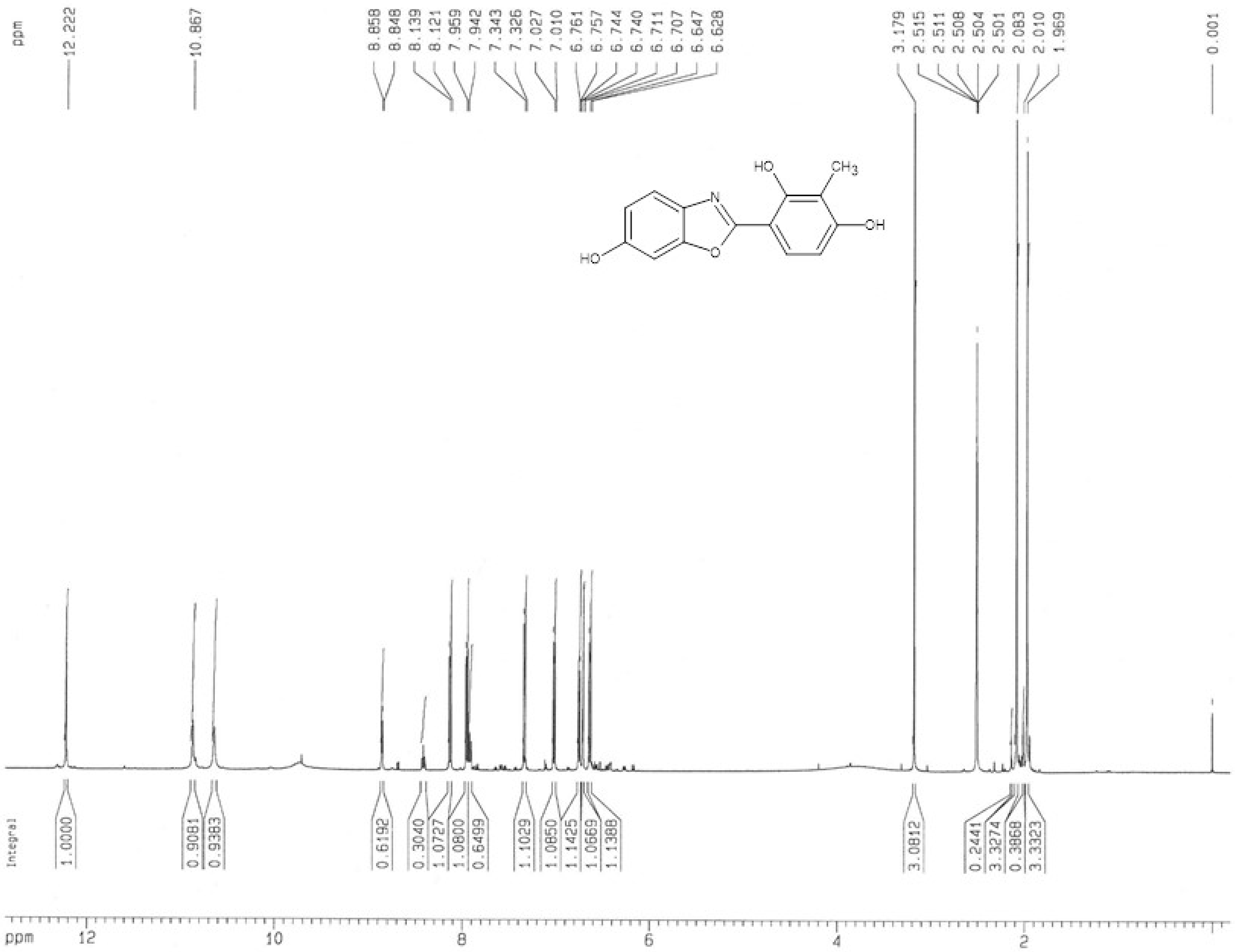

Current Data Parameters

EXPNO	1
PROCNO	1

F2 - Acquisition Parameters

Time	11.10
INSTRUM	spect
PROBHD	5 mm Dual 13
PULPROG	zg
TD	49152
SOLVENT	DMSO
NS	32
DS	0
SWH	10000.000 Hz
FIDRES	0.203451 Hz
AQ	2.4576499 sec
RG	256
DW	50.000 usec
DE	6.78 usec
TE	303.0 K
D1	1.50000000 sec

============ CHANNEL f1 ============

NUC1	1H
P1	4.00 usec
PL1	0.00 dB
SFO1	500.1330008 MHz

F2 - Processing parameters

SI	65536
SF	500.1300017 MHz
WDW	no
SSB	0
LB	0.00 Hz
GB	0
PC	8.00

1D NMR plot parameters

CX	22.00 cm
F1P	12.870 ppm
F1	6436.61 Hz
F2P	-0.191 ppm
F2	-95.64 Hz
PPMCM	0.59369 ppm/cm
HZCM	296.92084 Hz/cm

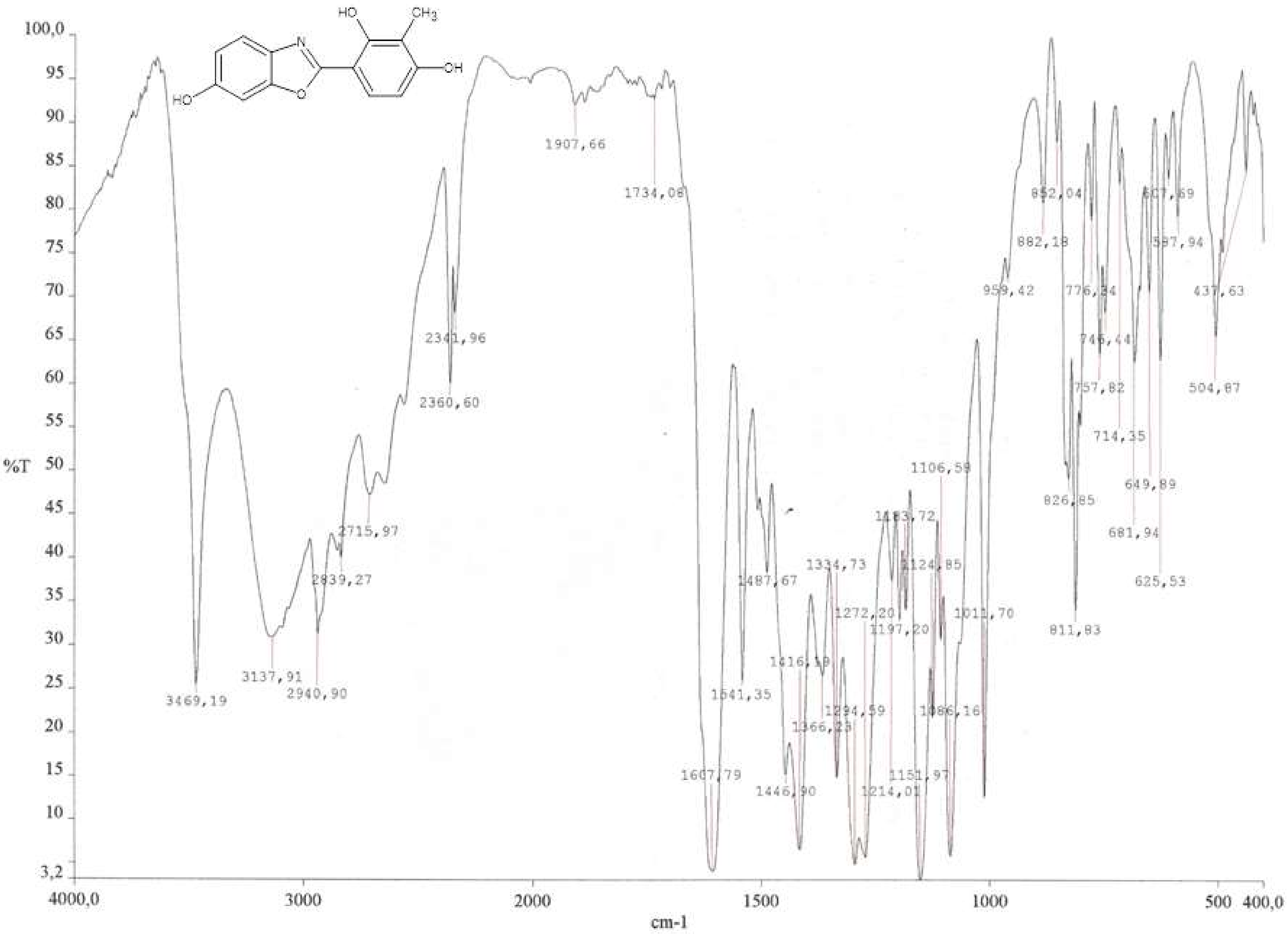

d:\pel_data\spectra\up834.sp - UNIWERSYTET PRZYRODNICZY LUBLIN

File Name	:	E:\Inne\LR_Lubl\an2120.ms2
File Type	:	Lo-Res Data - Ctd (Magnet)
File Source	:	Acquired on MASPEC II system [II32/99D9]
File Title	:	EI 70 eV 33-800
Operator	:	Małgorzata
Instrument	:	AMD 604

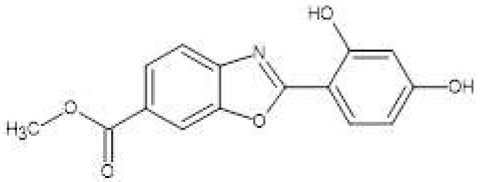

SCAN GRAPH. Flagging=Nominal M/z.
Scan 53#7:42. Entries=753. Base M/z=285.4. 100% Int.=91.0592. Temp =273.

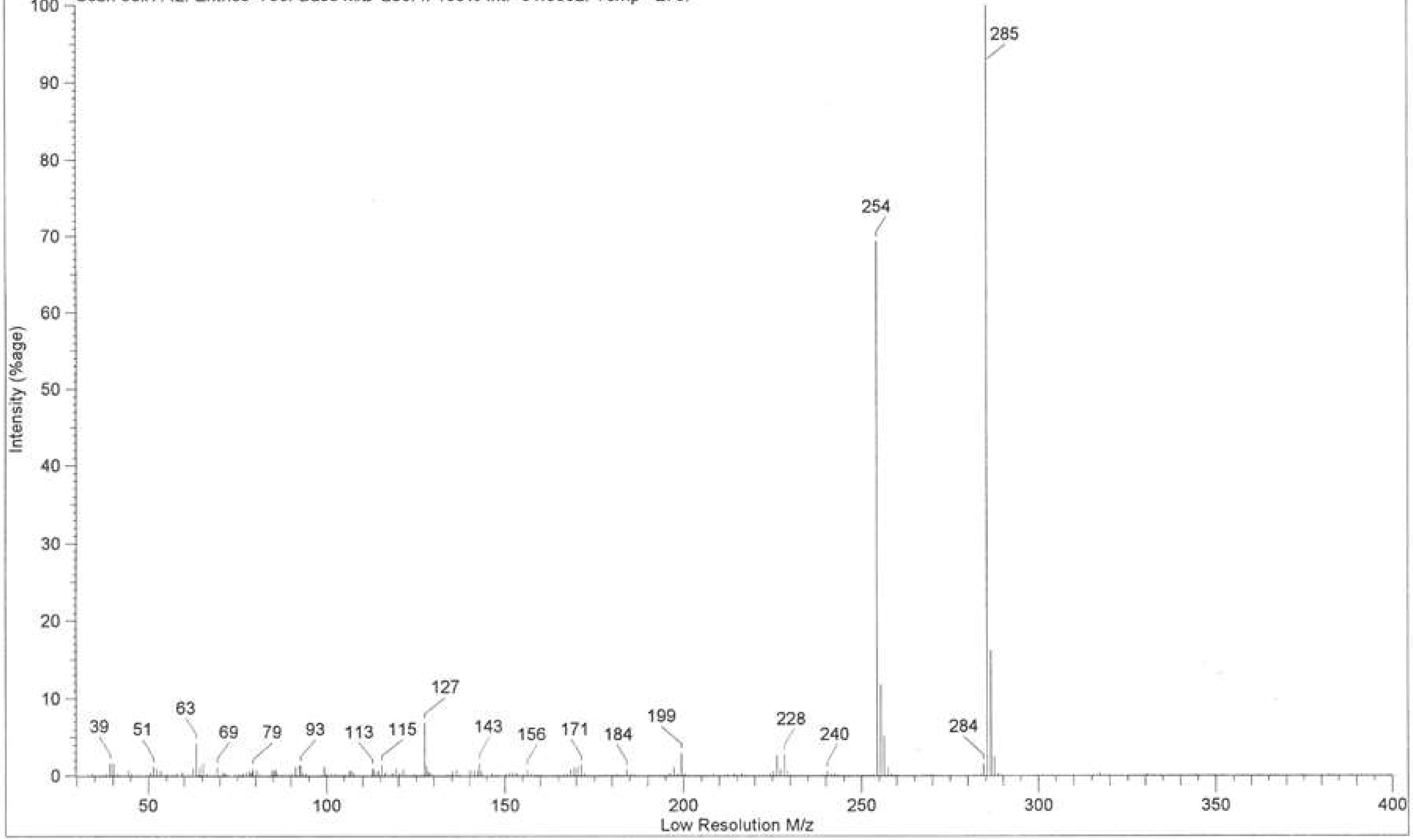

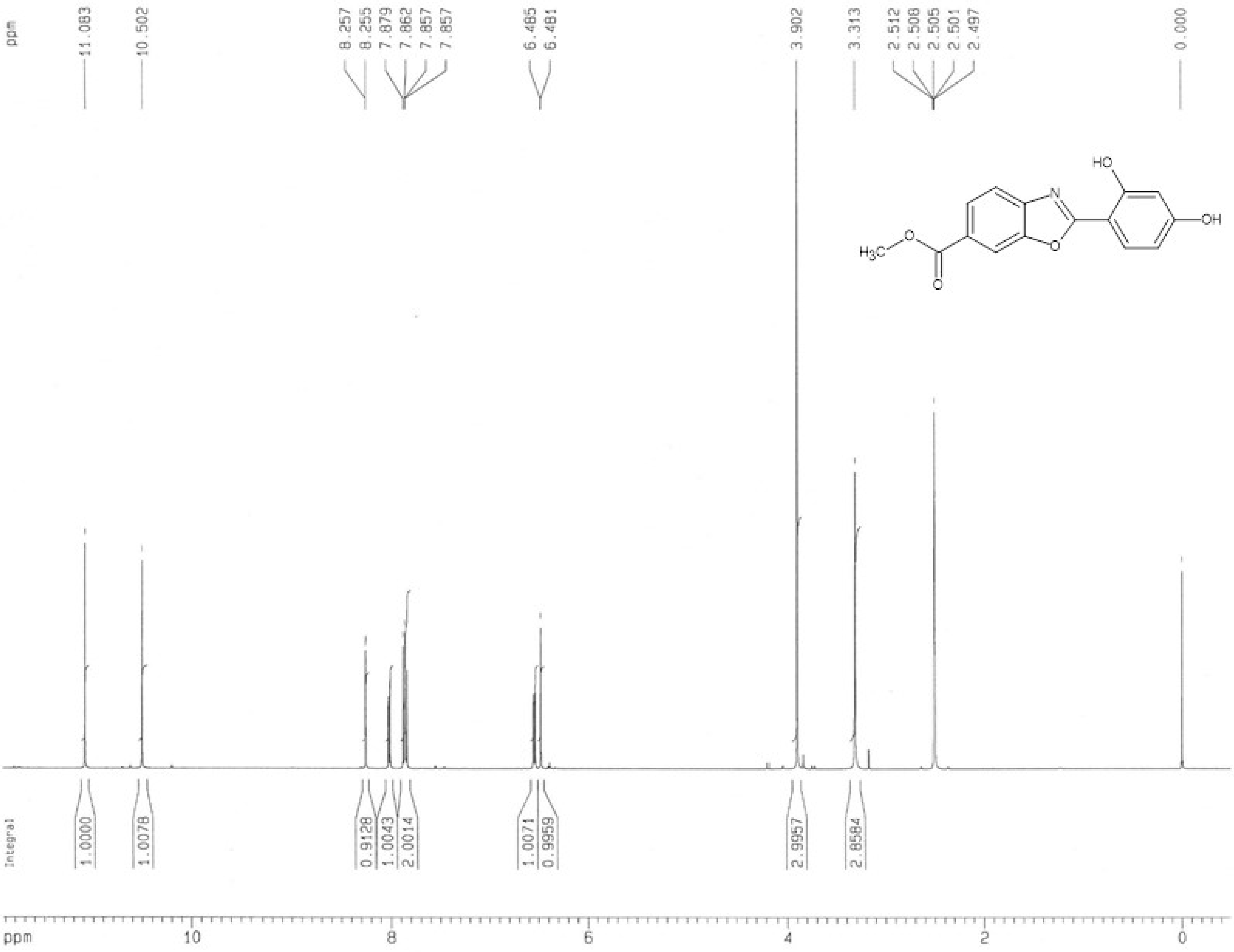

Current Data Parameters

EXPNO	1
PROCNO	1

F2 - Acquisition Parameters

Time	16.29
INSTRUM	spect
PROBHD	5 mm TBI 1H/1
PULPROG	zg
TD	49152
SOLVENT	DMSO
NS	32
DS	0
SWH	7645.260 Hz
FIDRES	0.155543 Hz
AQ	3.2145908 sec
RG	203.2
DW	65.400 usec
DE	6.78 usec
TE	303.0 K
D1	1.00000000 sec

============ CHANNEL f1 ============

NUC1	1H
P1	3.00 usec
PL1	3.00 dB
SFO1	500.1330057 MHz

F2 - Processing parameters

SI	65536
SF	500.1300032 MHz
WDW	EM
SSB	0
LB	0.00 Hz
GB	0
PC	0.30

1D NMR plot parameters

CX	22.00 cm
F1P	11.919 ppm
F1	5961.24 Hz
F2P	-0.493 ppm
F2	-246.71 Hz
PPMCM	0.56421 ppm/cm
HZCM	282.17960 Hz/cm

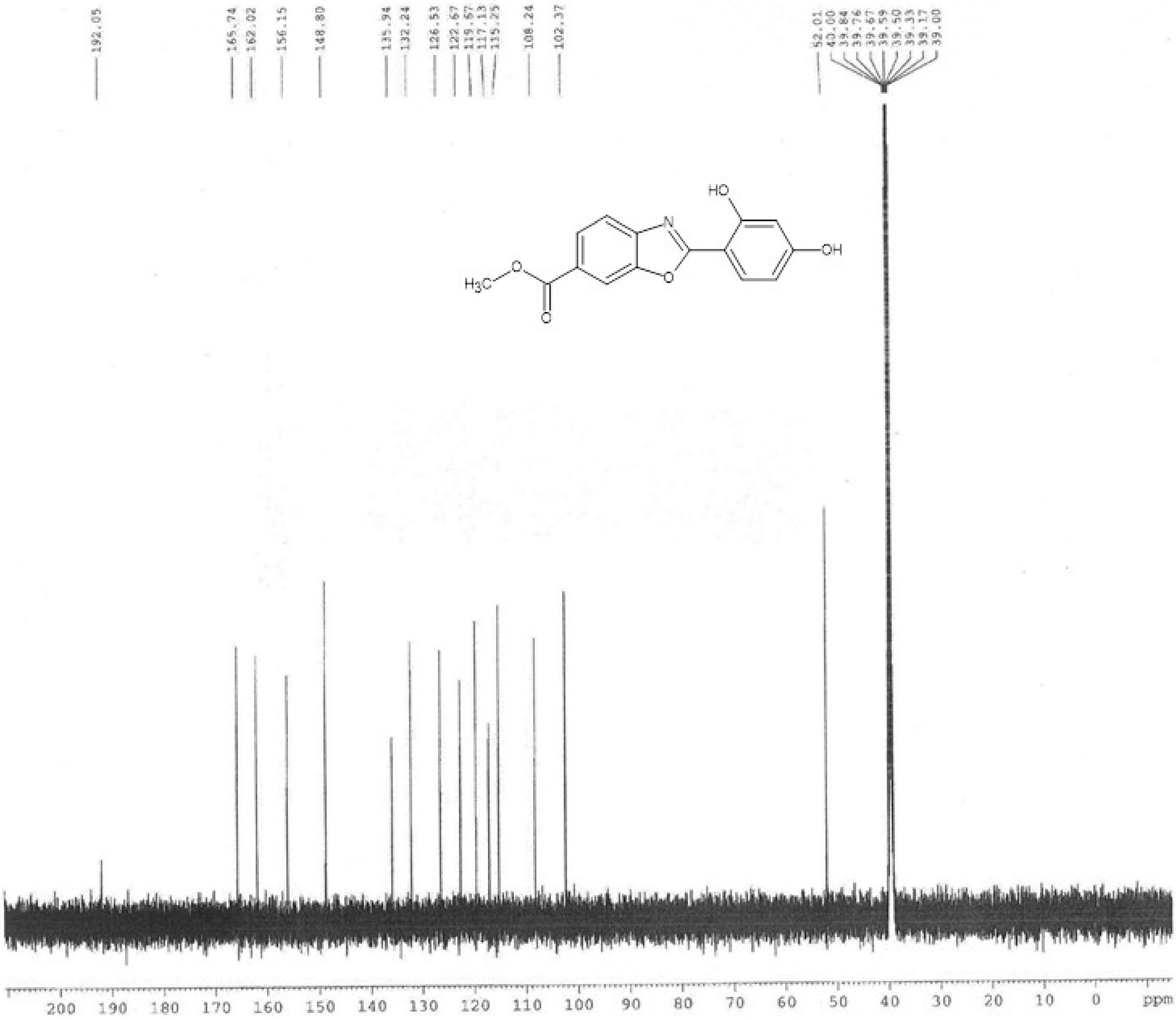

BRUKER

Current Data Parameters

EXPNO	2
PROCNO	1

F2 - Acquisition Parameters

Time	15.29
INSTRUM	DRX
PROBHD	5 mm TBI 1H/13
PULPROG	zgpg
TD	65536
SOLVENT	CDCl3
NS	572
DS	4
SWH	32679.738 Hz
FIDRES	0.498653 Hz
AQ	1.0027508 sec
RG	32768
DW	15.300 usec
DE	7.10 usec
TE	303.0 K
D1	1.00000000 sec
d11	0.03000000 sec
DELTA	0.89999998 sec
TD0	1

======== CHANNEL f1 ========

NUC1	13C
P1	5.00 usec
PL1	-3.00 dB
SFO1	125.7703643 MHz

======== CHANNEL f2 ========

CPDPRG2	waltz16
NUC2	1H
PCPD2	98.00 usec
PL2	3.00 dB
PL12	23.00 dB
PL13	32.00 dB
SFO2	500.1320005 MHz

F1 - Acquisition parameters

ND0	1
TD	128
SFO1	500.132 MHz
FIDRES	7.812500 Hz
SW	1.999 ppm
FnMODE	QF

F2 - Processing parameters

SI	262144
SF	125.7578525 MHz
WDW	EM
SSB	0
LB	0.50 Hz
GB	0
PC	1.40

F1 - Processing parameters

SI	1024
MC2	QF
SF	500.1300000 MHz
WDW	SINE
SSB	0
LB	0.30 Hz
GB	0.1

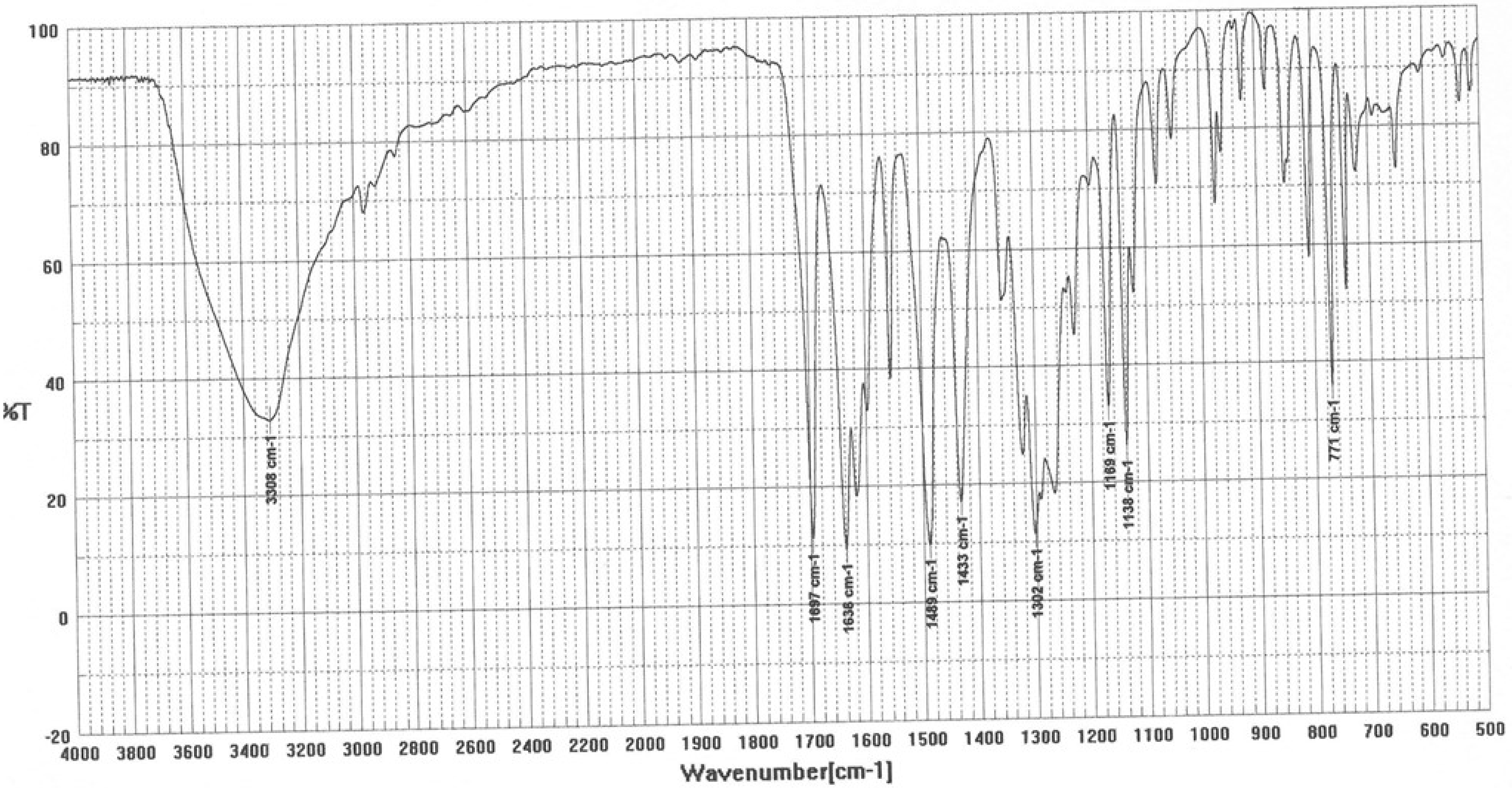

Sample	0,5mg/270mg KBr
Resolution	1 cm-1
Accumulation	30
Apodization	Cosine

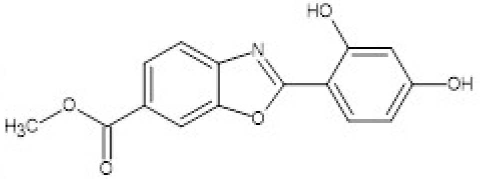

File Name : E:\Inne\LR_Lubl\an3915.ms2

File Type : Lo-Res Data - Ctd (Magnet)
File Source : Acquired on MASPEC II system [II32/99D9]
File : 4 EI 70 eV 33-800
Operator : Małgorzata
Instrument : AMD 604

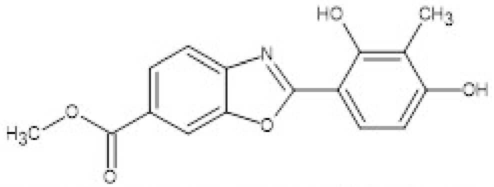

SCAN GRAPH. Flagging=Nominal M/z.
Scan 79#11:33. Entries=957. Base M/z=299.2. 100% Int.=43.6736. Temp =223.

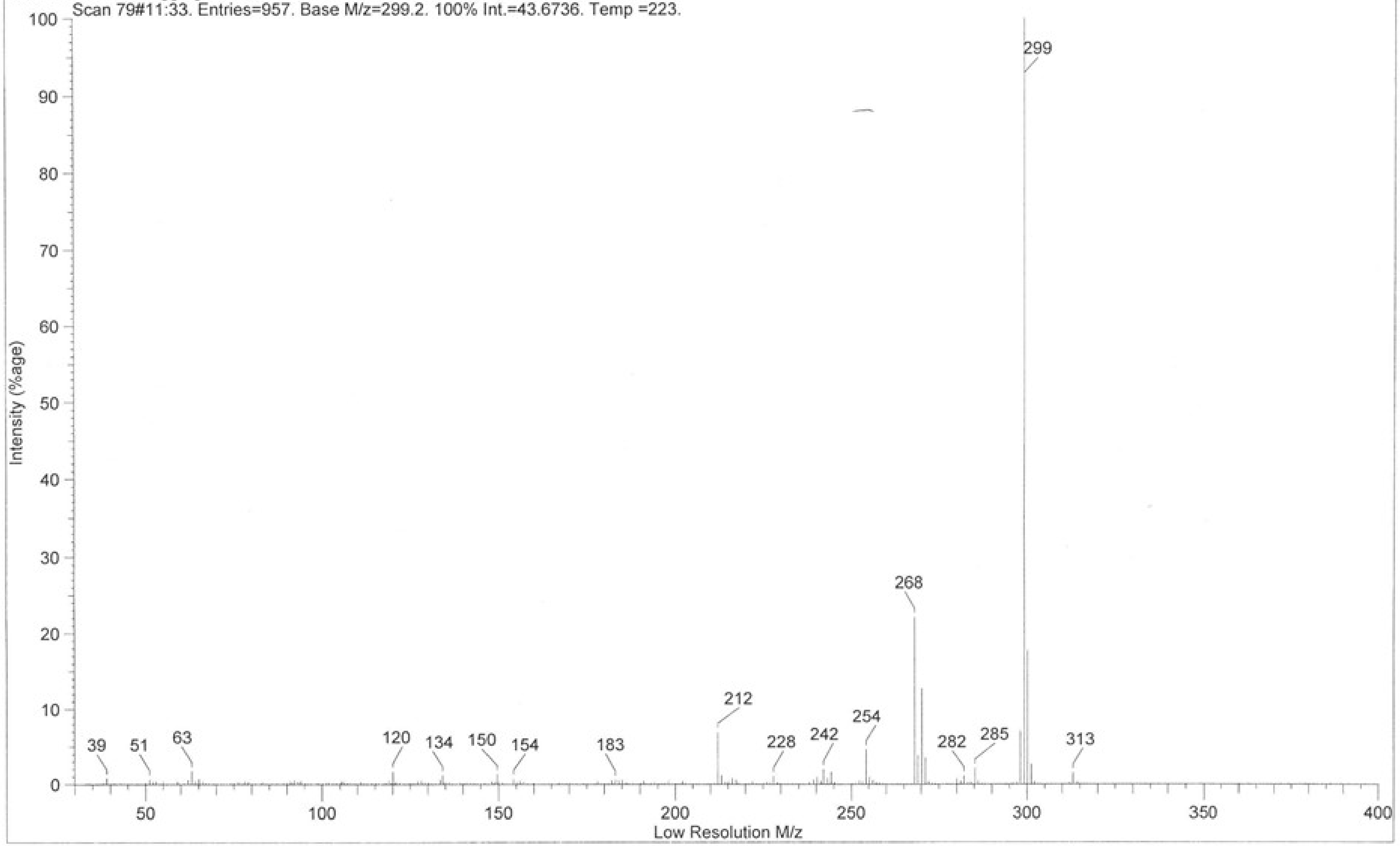

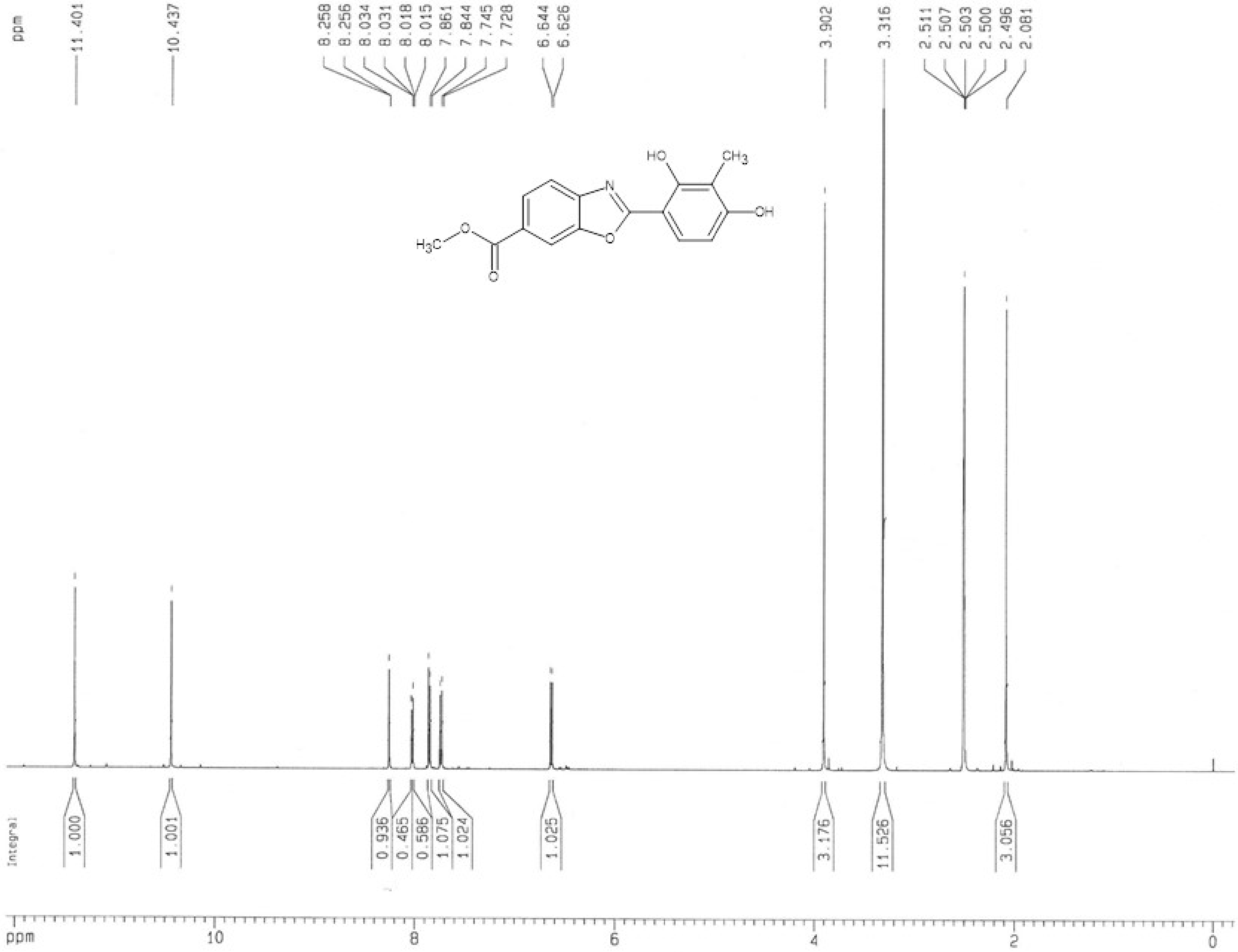

Current Data Parameters

EXPNO	1
PROCNO	1

F2 - Acquisition Parameters

Time	9.49
INSTRUM	spect
PROBHD	5 mm TBI 1H/1
PULPROG	zg
TD	49152
SOLVENT	DMSO
NS	32
DS	0
SWH	10000.000 Hz
FIDRES	0.203451 Hz
AQ	2.4576499 sec
RG	256
DW	50.000 usec
DE	6.78 usec
TE	303.0 K
D1	1.00000000 sec

============ CHANNEL f1 ==========

NUC1	1H
P1	3.00 usec
PL1	3.00 dB
SFO1	500.1330008 MHz

F2 - Processing parameters

SI	65536
SF	500.1300043 MHz
WDW	no
SSB	0
LB	0.00 Hz
GB	0
PC	8.00

1D NMR plot parameters

CX	22.00 cm
F1P	12.085 ppm
F1	6044.23 Hz
F2P	-0.219 ppm
F2	-109.45 Hz
PPMCM	0.55928 ppm/cm
HZCM	279.71274 Hz/cm

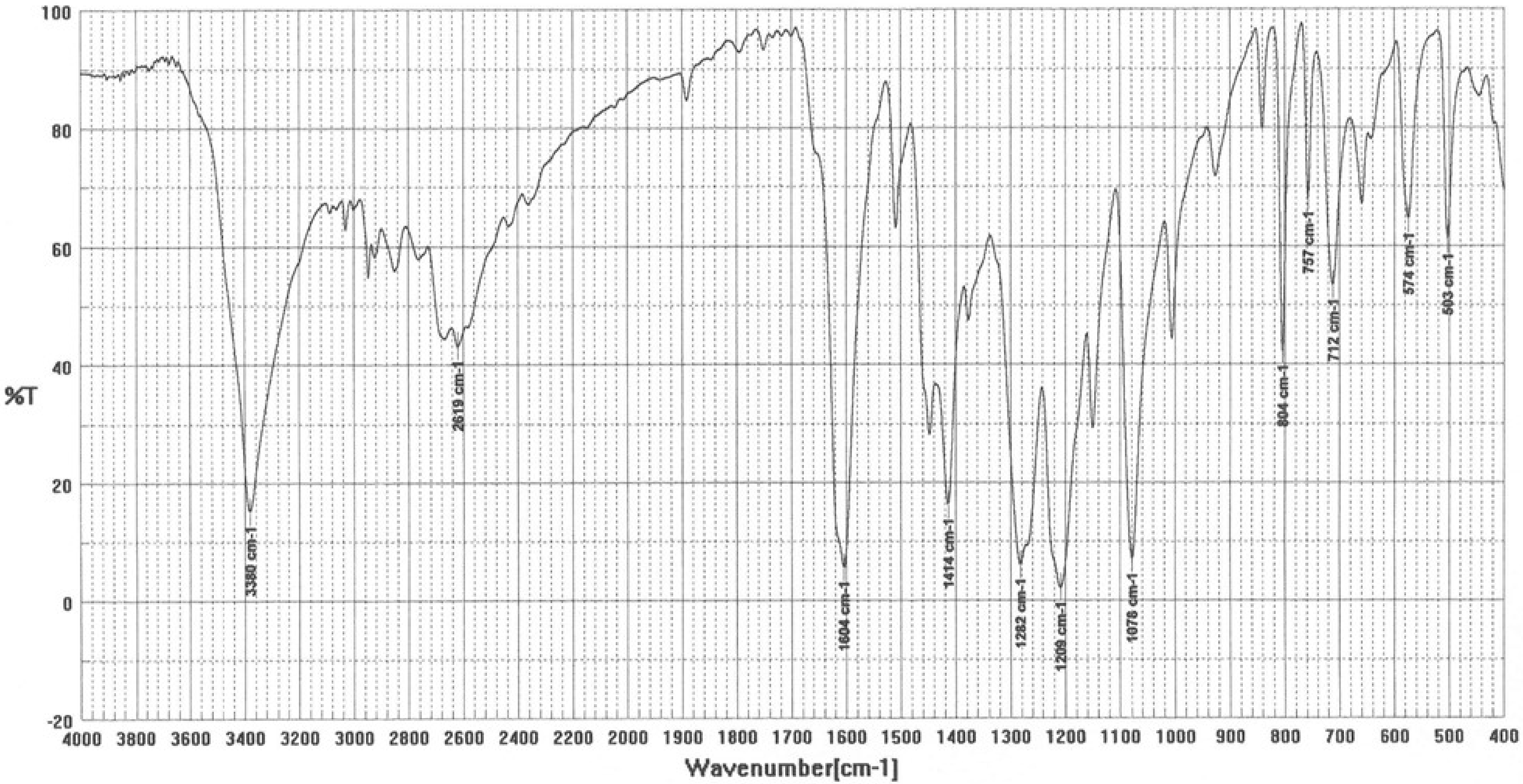

Sample Name	4 (1,0mg/270mg KBr)
Resolution	1 cm-1
Accumulation	30
Apodization	Cosine

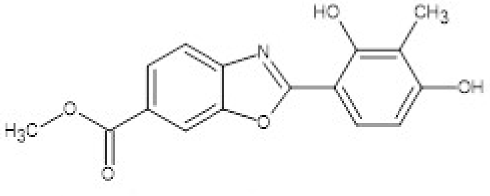

File Name	:	E:\Inne\LR_Lubl\an4454.ms2
File Type	:	Lo-Res Data - Ctd (Magnet)
File Source	:	Acquired on MASPEC II system [II32/99D9]
File Title	:	5 (EI 70 eV 33-800)
Operator	:	Małgorzata
Instrument	:	AMD 604

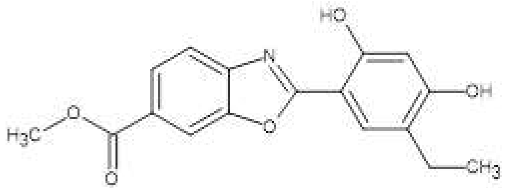

SCAN GRAPH. Flagging=Nominal M/z.

Scan 44#6:29. Entries=684. Base M/z=298.1. 100% Int.=63.0016. Temp =188.

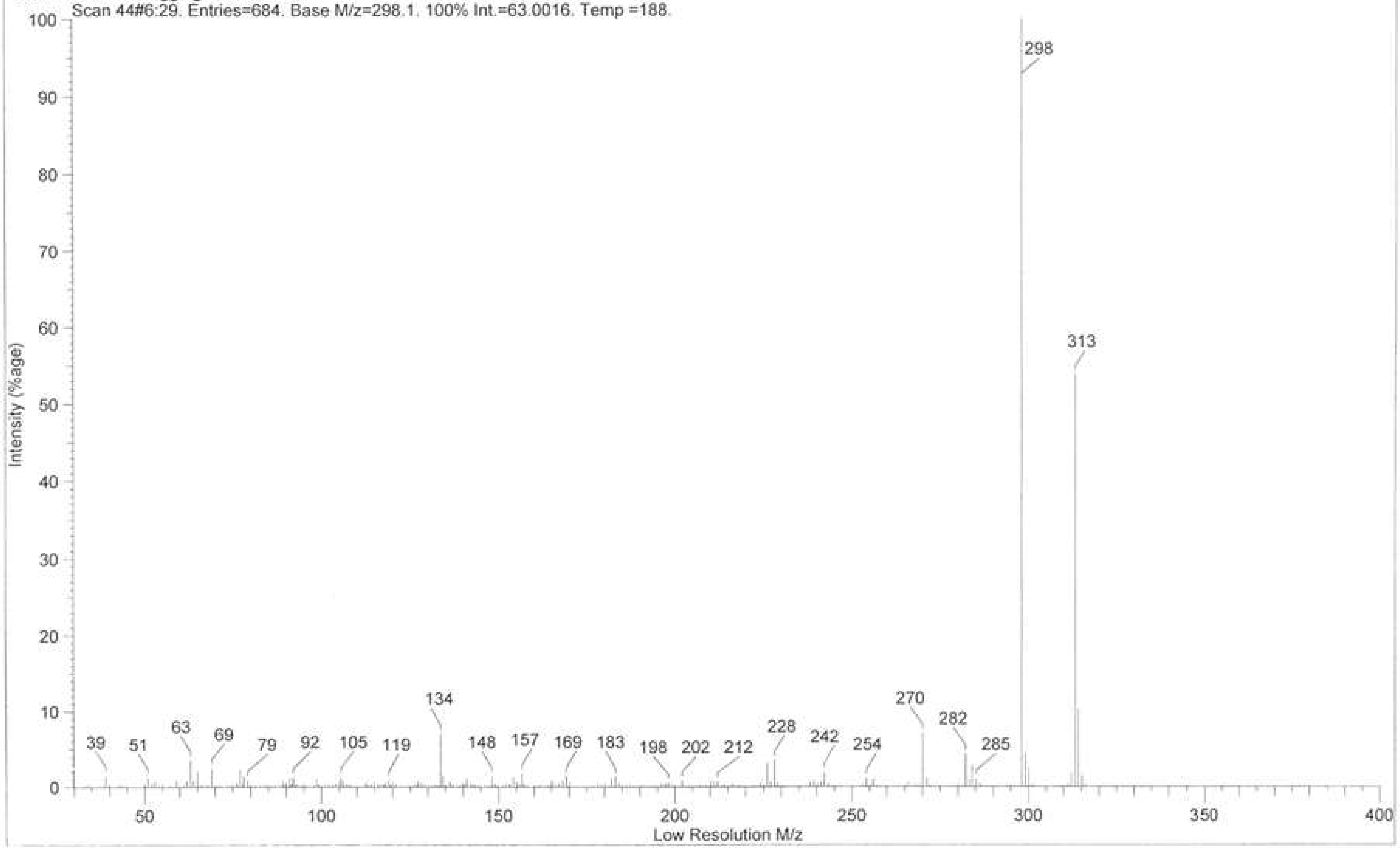

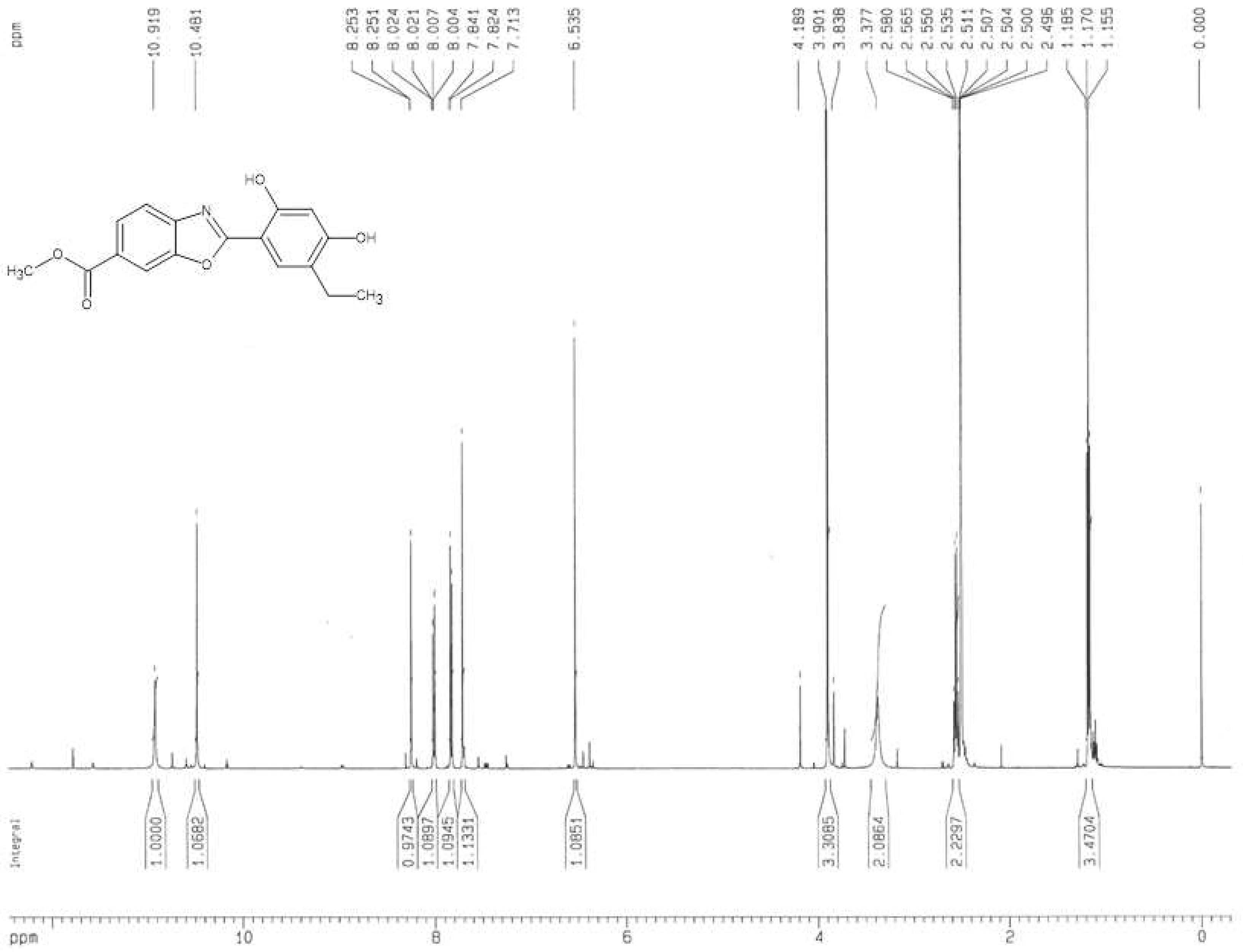

Current Data Parameters

EXPNO	1
PROCNO	1

F2 - Acquisition Parameters

Time	20.14
INSTRUM	spect
PROBHD	5 mm TBI 1H/1
PULPROG	zg
TD	49152
SOLVENT	DMSO
NS	32
DS	0
SWH	8012.820 Hz
FIDRES	0.163021 Hz
AQ	3.0671349 sec
RG	203.2
DW	62.400 usec
DE	6.78 usec
TE	303.0 K
D1	1.00000000 sec

============ CHANNEL f1 ============

NUC1	1H
P1	3.00 usec
PL1	3.00 dB
SFO1	500.1335009 MHz

F2 - Processing parameters

SI	65536
SF	500.1300036 MHz
WDW	no
SSB	0
LB	0.00 Hz
GB	0
PC	8.00

1D NMR plot parameters

CX	22.00 cm
F1P	12.453 ppm
F1	6228.23 Hz
F2P	-0.314 ppm
F2	-156.99 Hz
PPMCM	0.58032 ppm/cm
HZCM	290.23712 Hz/cm

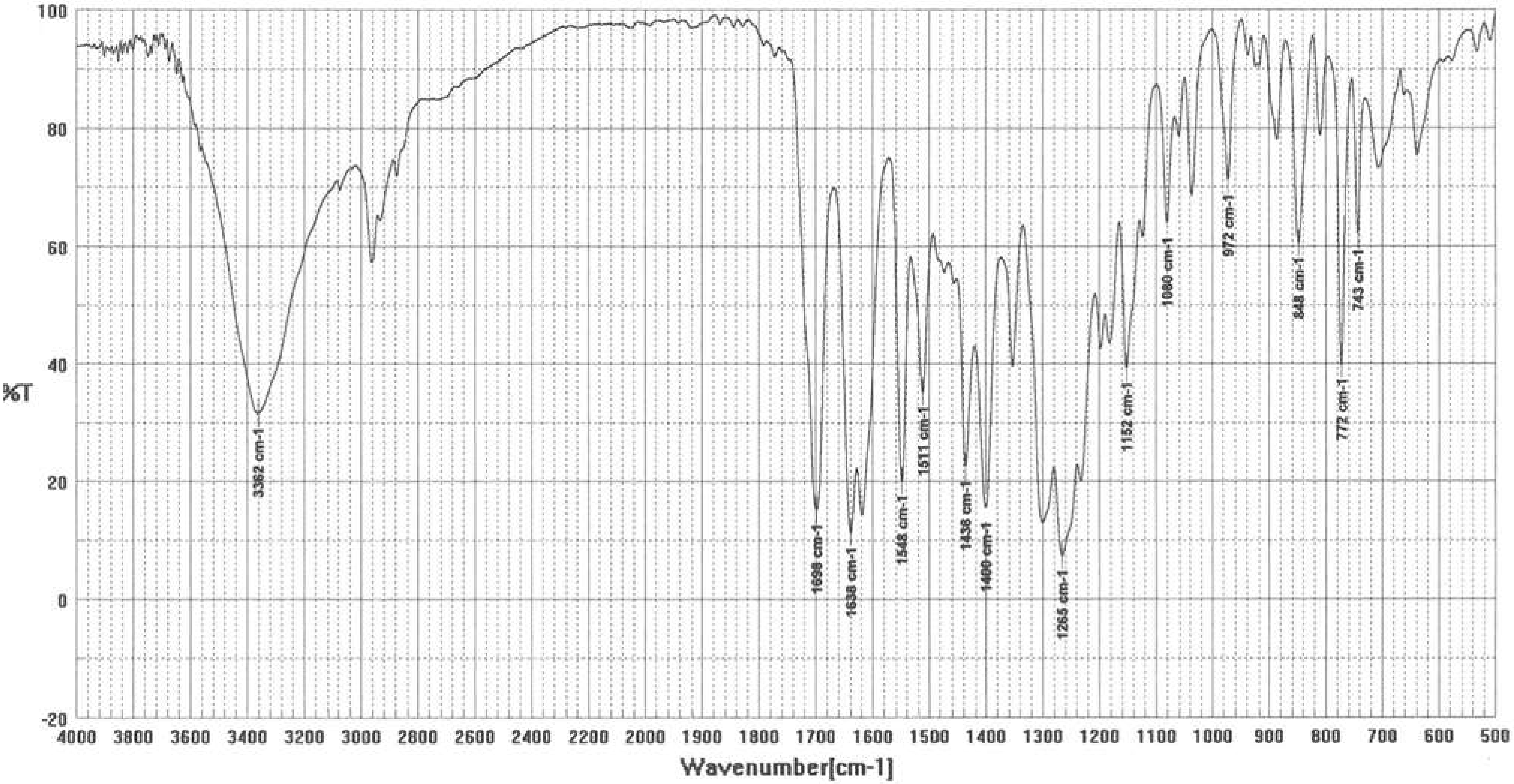

Sample Name	5 (0,8mg/270mg KBr)
Resolution	1 cm-1
Accumulation	30
Apodization	Cosine

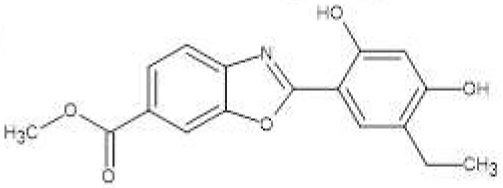

File Name	:	E:\Inne\LR_Lubl\an3917.ms2
File Type	:	Lo-Res Data - Ctd (Magnet)
File Source	:	Acquired on MASPEC II system [II32/99D9]
File Title	:	6 (EI 70 eV 33-800)
Operator	:	Malgorzata
Instrument	:	AMD 604

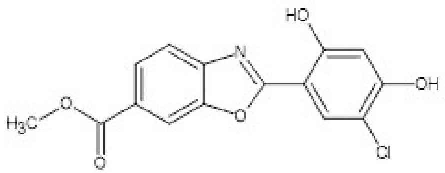

SCAN GRAPH. Flagging=Nominal M/z.

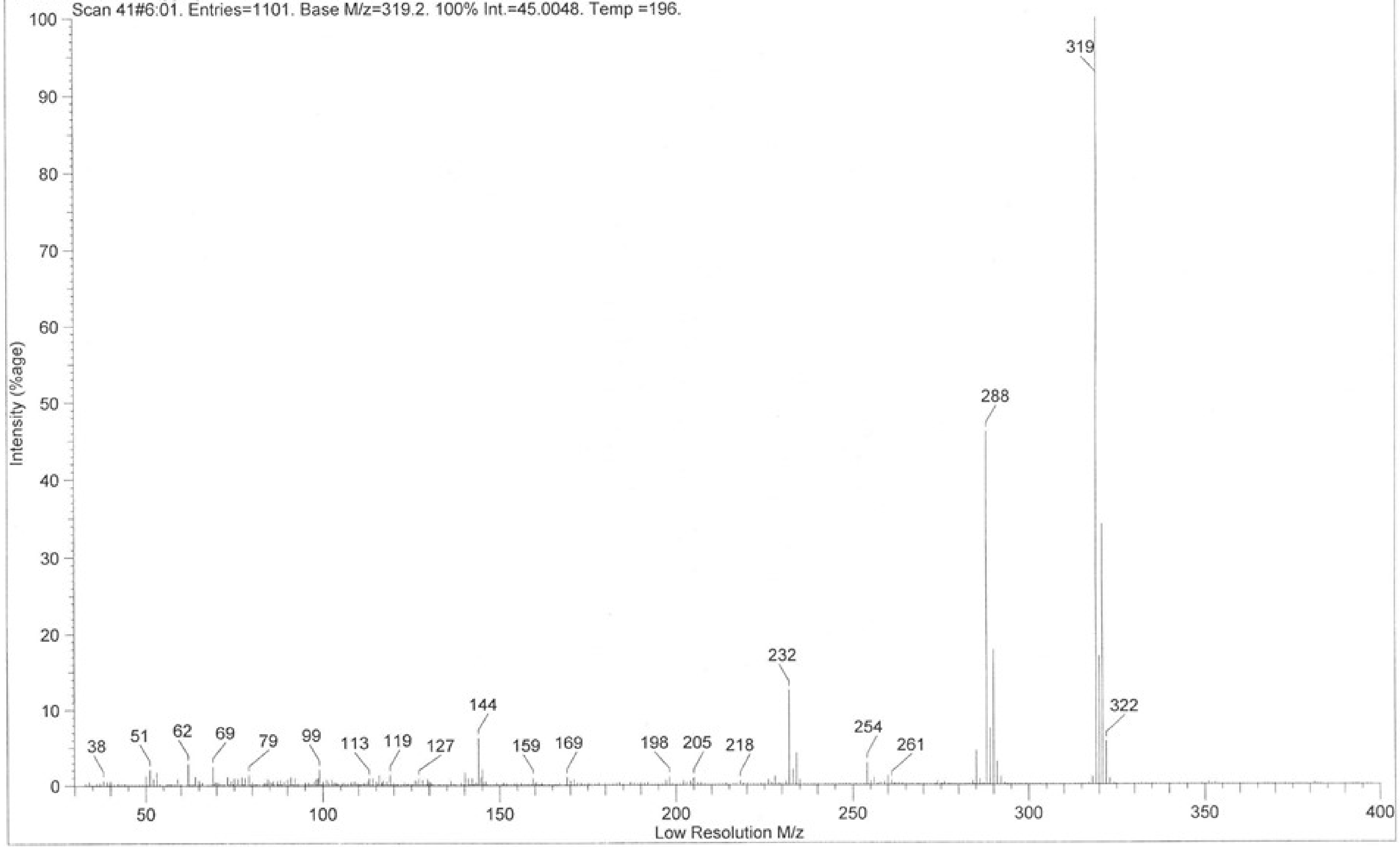

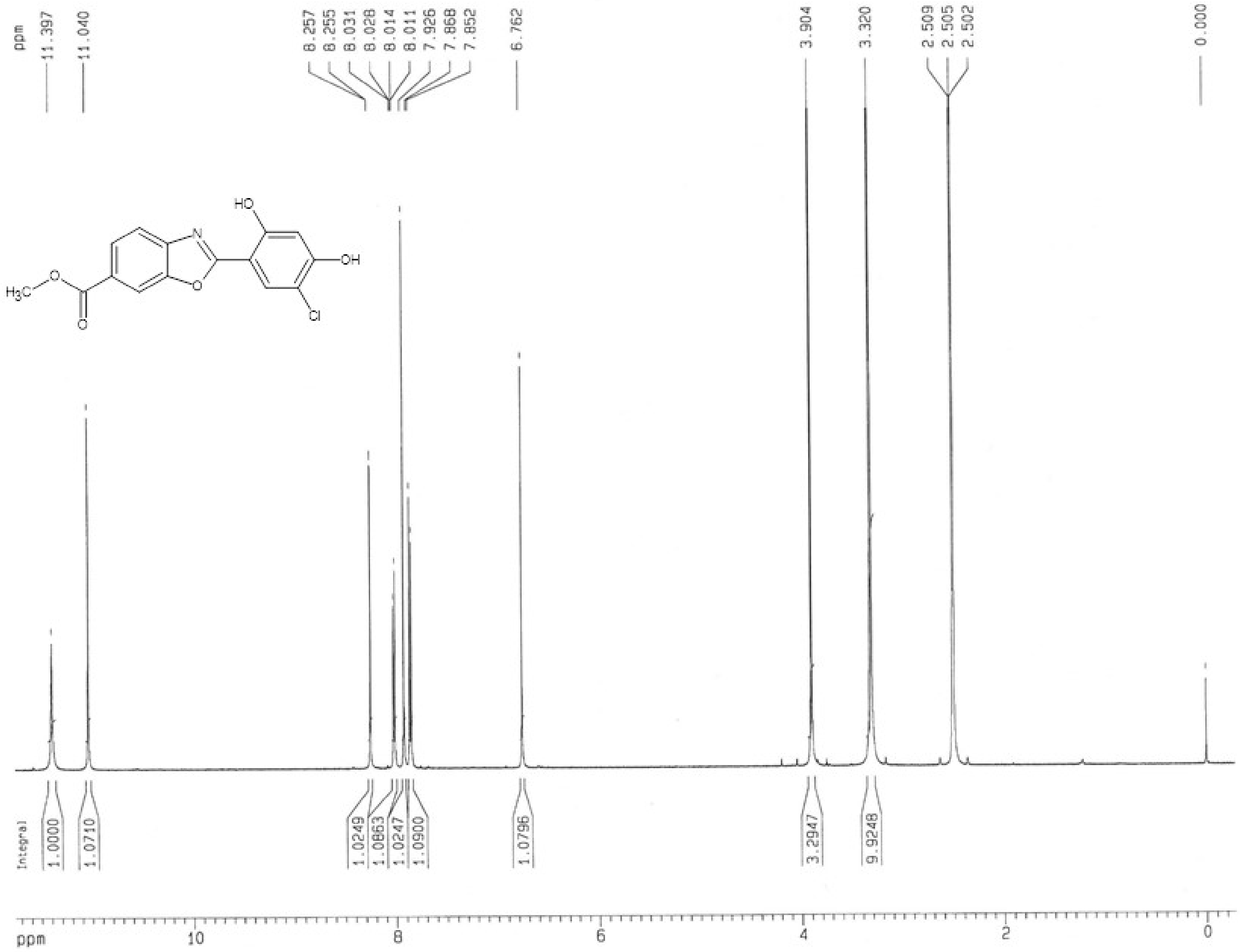

Current Data Parameters

EXPNO	1
PROCNO	1

F2 - Acquisition Parameters

Time	10.48
INSTRUM	spect
PROBHD	5 mm TBI 1H/1
PULPROG	zg
TD	49152
SOLVENT	DMSO
NS	32
DS	0
SWH	8992.806 Hz
FIDRES	0.182959 Hz
AQ	2.7329011 sec
RG	456.1
DW	55.600 usec
DE	6.78 usec
TE	303.0 K
D1	1.00000000 sec

============ CHANNEL f1 ============

NUC1	1H
P1	3.00 usec
PL1	3.00 dB
SFO1	500.1340010 MHz

F2 - Processing parameters

SI	65536
SF	500.1300036 MHz
WDW	no
SSB	0
LB	0.00 Hz
GB	0
PC	8.00

1D NMR plot parameters

CX	22.00 cm
F1P	11.759 ppm
F1	5880.88 Hz
F2P	-0.282 ppm
F2	-141.09 Hz
PPMCM	0.54731 ppm/cm
HZCM	273.72580 Hz/cm

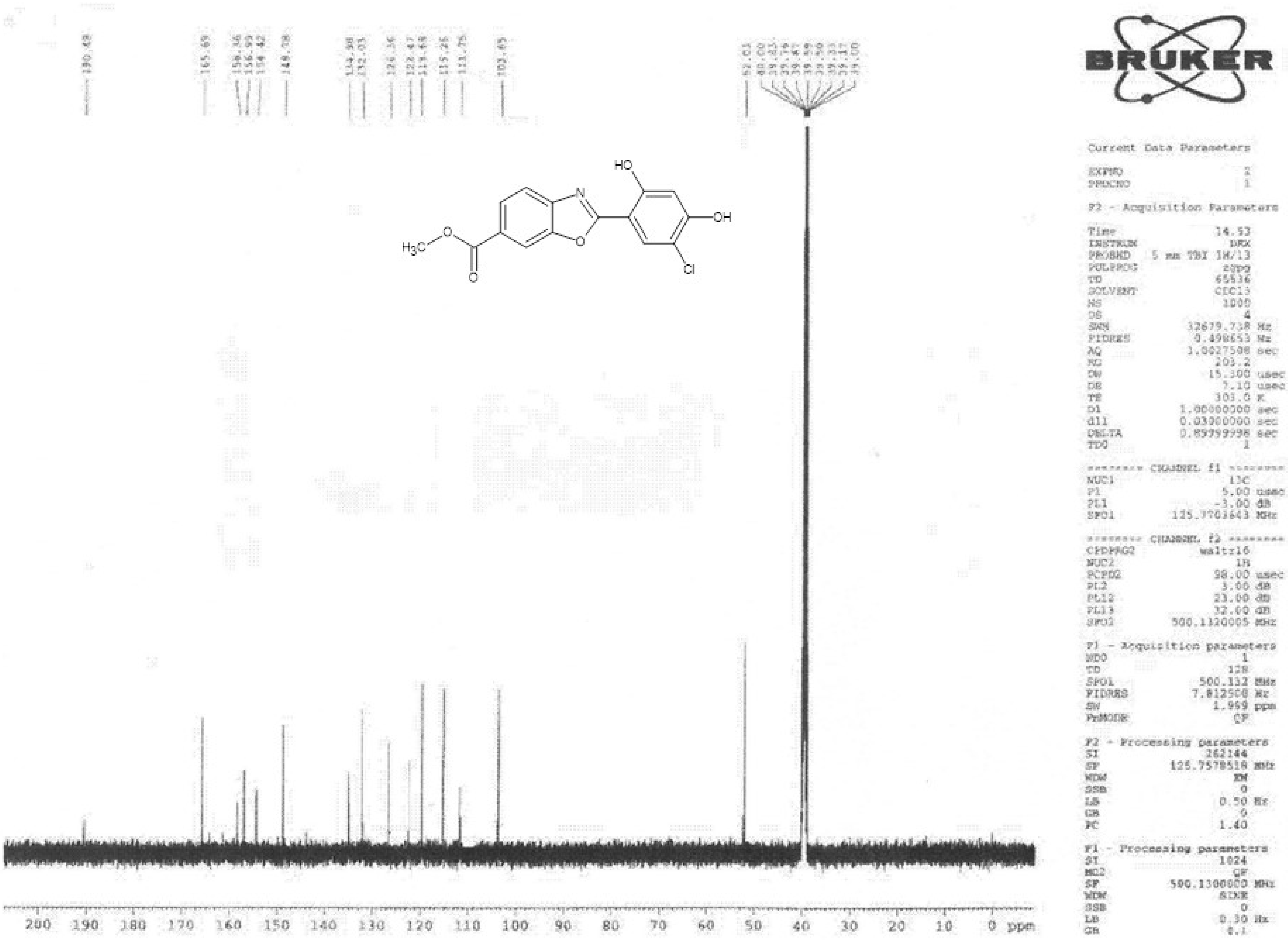
190.43
165.69
158.36
156.90
154.42
148.98
134.98
132.03
126.36
122.47
119.68
115.26
111.75
103.65
52.03
HO
OH
N
O
H_3C
O
O
Cl
200 190 180 170 160 150 140 130 120 110 100 90 80 70 60 50 40 30 20 10 0 ppm
BRUKER
Current Data Parameters
EXPNO 2
PROCNO 1
F2 - Acquisition Parameters
Time 14.53
INSTRUM DRX
PROBHD 5 mm TBI 1H/13
PULPROG zgpg
TD 65536
SOLVENT CDCl3
NS 1000
DS 4
SWH 32679.738 Hz
FIDRES 0.498653 Hz
AQ 1.0027508 sec
RG 203.2
DW 15.300 usec
DE 7.10 usec
TE 303.0 K
D1 1.00000000 sec
d11 0.03000000 sec
DELTA 0.89999998 sec
TD0 1
======== CHANNEL f1 ========
NUC1 13C
P1 5.00 usec
PL1 -3.00 dB
SFO1 125.7703643 MHz
======== CHANNEL f2 ========
CPDPRG2 waltz16
NUC2 1H
PCPD2 98.00 usec
PL2 3.00 dB
PL12 23.00 dB
PL13 32.00 dB
SFO2 500.1120005 MHz
F1 - Acquisition parameters
ND0 1
TD 128
SFO1 500.132 MHz
FIDRES 7.812500 Hz
SW 1.969 ppm
FnMODE QF
F2 - Processing parameters
SI 262144
SF 125.7578518 MHz
WDW EM
SSB 0
LB 0.50 Hz
GB 0
PC 1.40
F1 - Processing parameters
SI 1024
MC2 QF
SF 500.1300000 MHz
WDW SINE
SSB 0
LB 0.30 Hz
GB 0.1

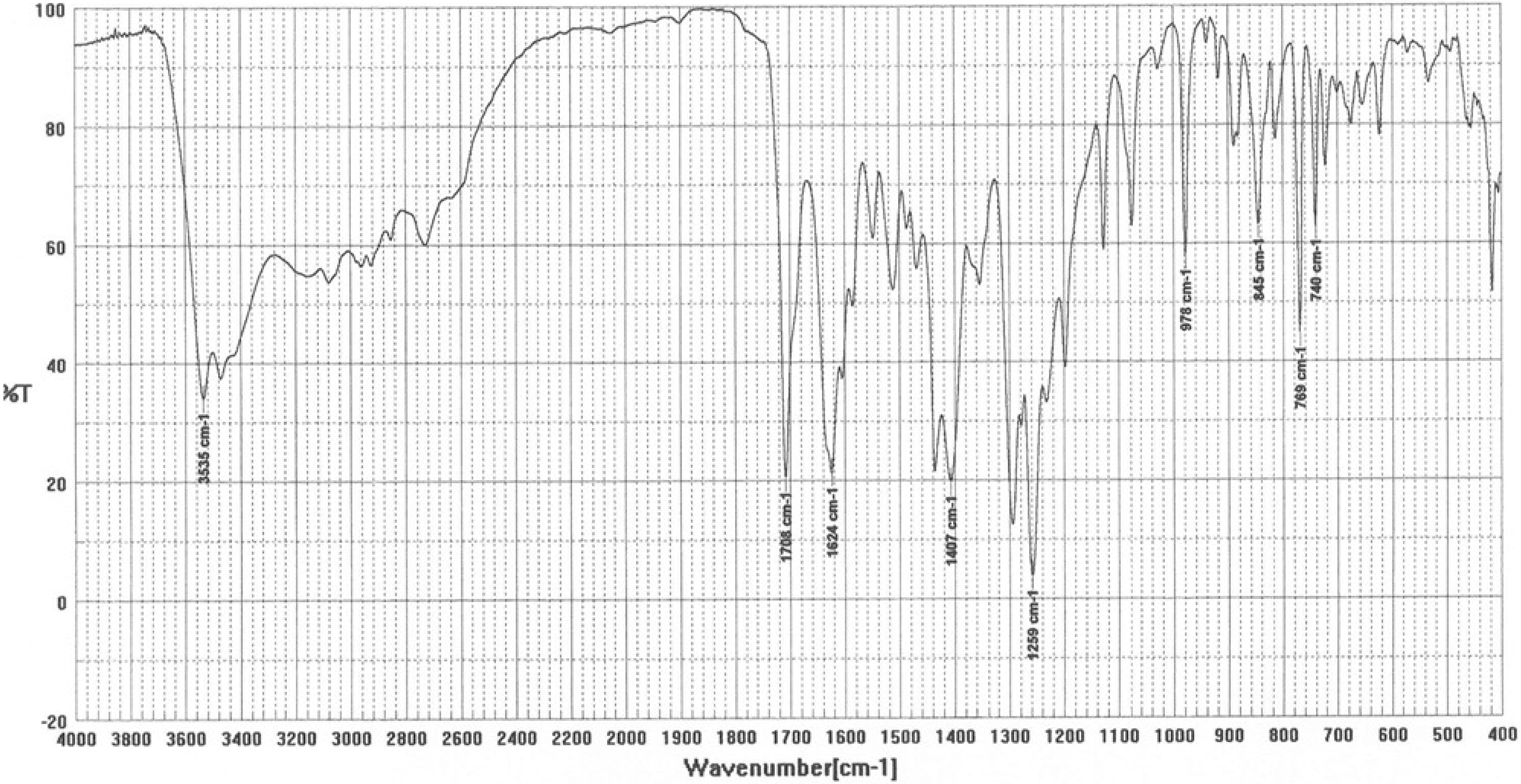

Sample Name	6 (0,8mg/270mg KBr)
Resolution	1 cm-1
Accumulation	30
Apodization	Cosine

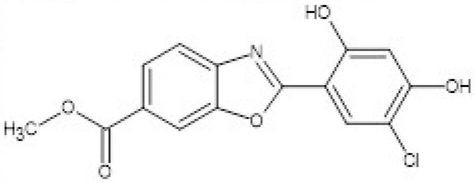

File Name	:	E:\Inne\LR_Lubl\an3920.ms2
File Type	:	Lo-Res Data - Ctd (Magnet)
File Source	:	Acquired on MASPEC II system [II32/99D9]
File Title	:	7 (EI 70 eV 33-800)
Operator	:	Małgorzata
Instrument	:	AMD 604

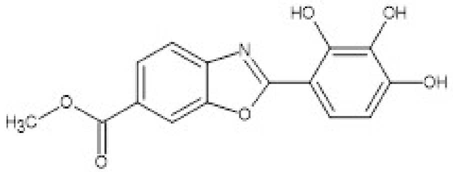

SCAN GRAPH. Flagging=Nominal M/z.

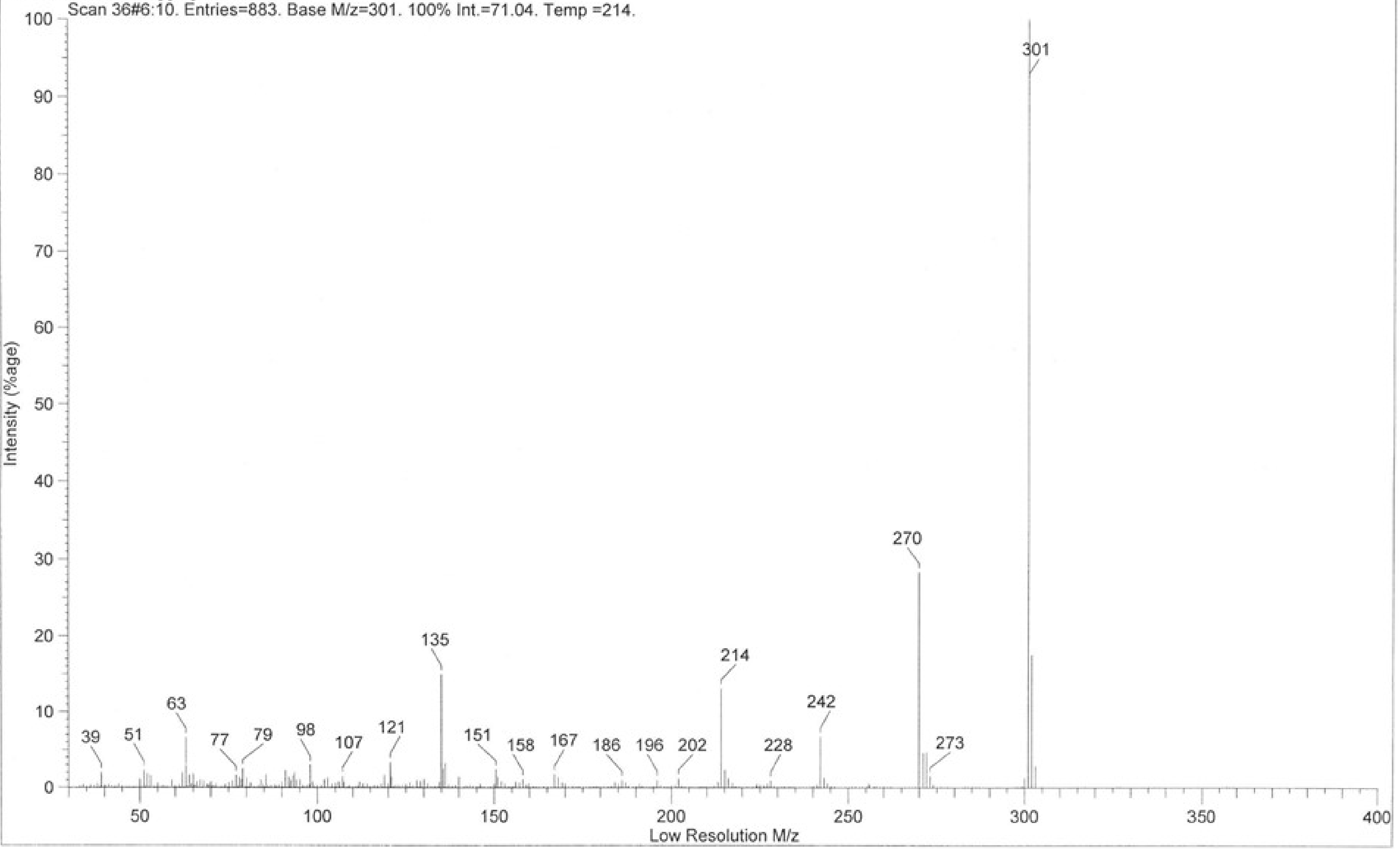

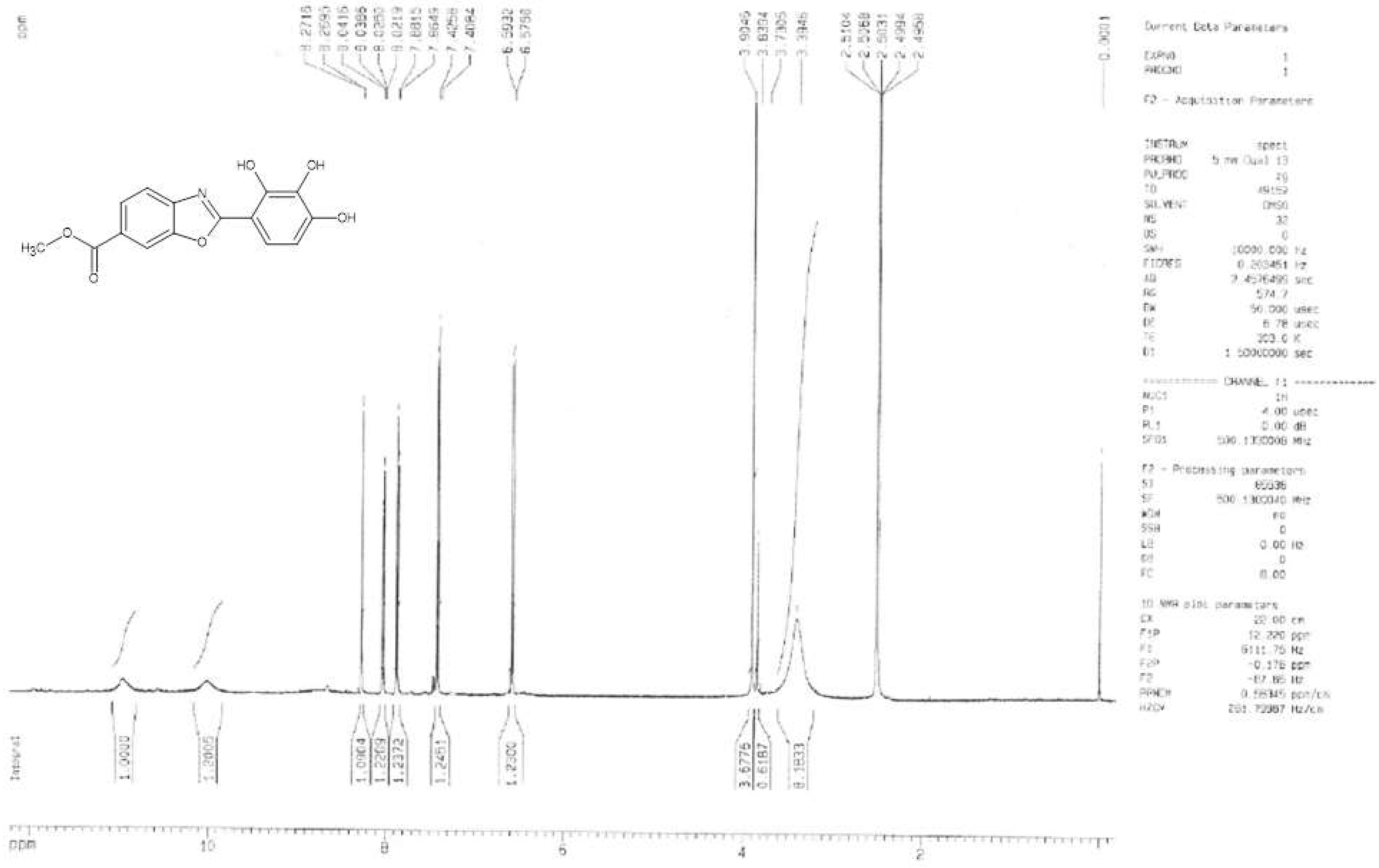

HO
OH
OH
N
O
H_3C
O
O
8.2716
8.2595
8.0416
8.0386
8.0250
8.0219
7.8815
7.8649
7.4258
7.4084
6.5932
6.5758
3.9046
3.8394
3.7305
3.3946
2.5104
2.5068
2.5031
2.4994
2.4958
0.0001
Integral
1.0000
1.3005
1.0904
1.2269
1.2372
1.2451
1.2300
3.6776
0.6167
8.1833
ppm
10
8
6
4
2
Current Data Parameters
EXPNO 1
PROCNO 1
F2 - Acquisition Parameters
INSTRUM spect
SOLVENT DMSO
NS 32
DS 0
SWH 10000.000 Hz
RG 574.7
DW 50.000 usec
DE 6.78 usec
D1 1.50000000 sec
CHANNEL f1
NUC1 1H
P1 4.00 usec
PL1 0.00 dB
F2 - Processing parameters
SI 65536
SSB 0
LB 0.00 Hz
GB 0
PC 8.00
1D NMR plot parameters
CX 22.00 cm
F1P 12.220 ppm
F1 6111.75 Hz
F2P -0.176 ppm
F2 -87.86 Hz

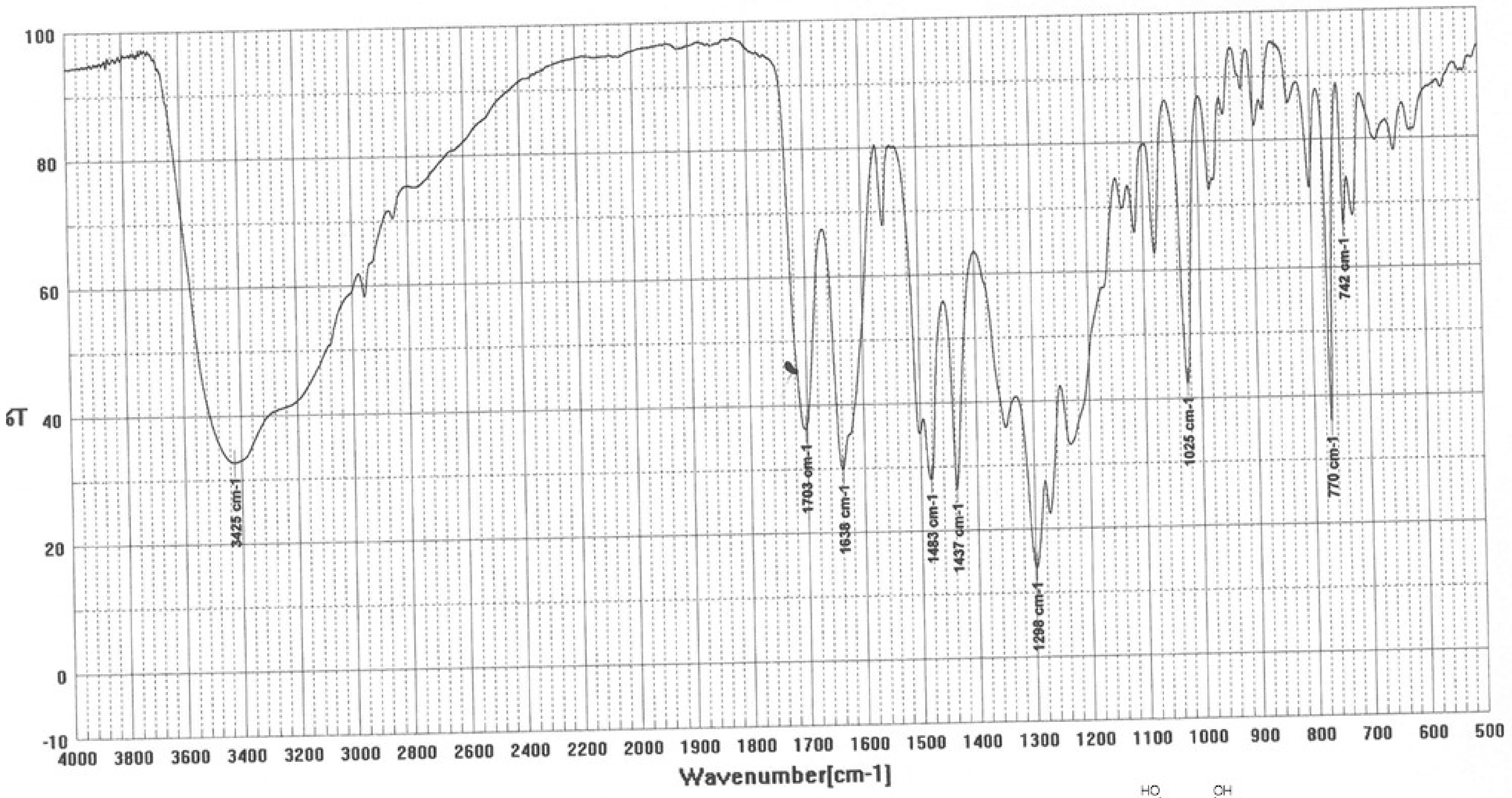

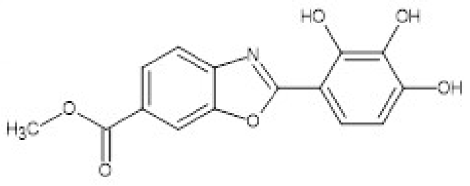

Sample Name	7 (0,7mg/270mg KBr)
Resolution	1 cm-1
Accumulation	30
Apodization	Cosine

File Name	:	E:\Inne\LR_Lubl\an3933.ms2
File Type	:	Lo-Res Data - Ctd (Magnet)
File Source	:	Acquired on MASPEC II system [II32/99D9]
File Title	:	8 (EI 70 eV 33-800)
Operator	:	Malgorzata
Instrument	:	AMD 604

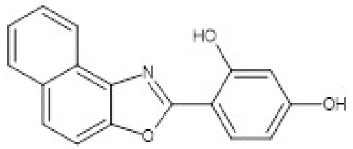

SCAN GRAPH. Flagging=Nominal M/z.

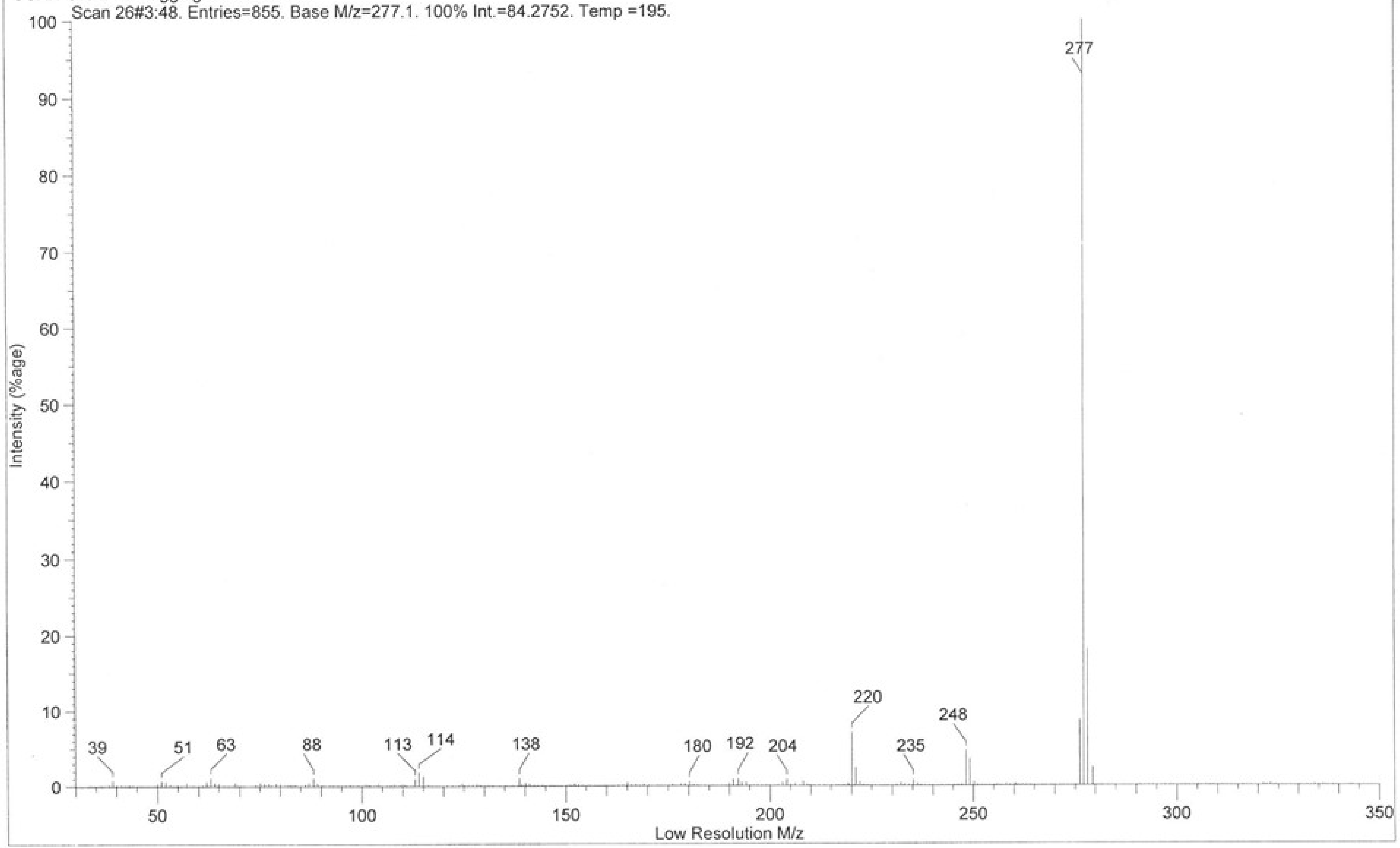

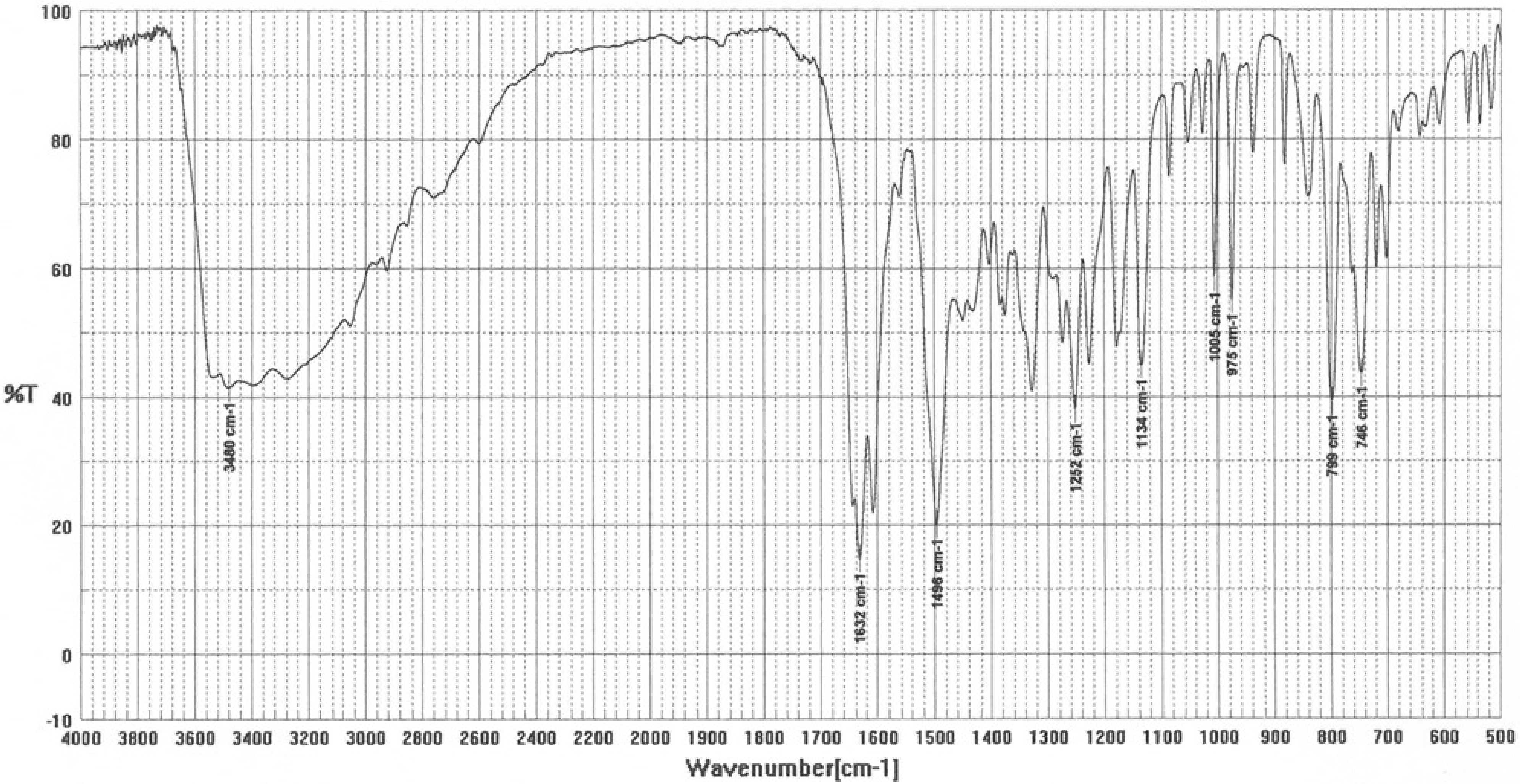

Sample Name	8 (0,8mg/270mg KBr)
Resolution	1 cm-1
Accumulation	30
Apodization	Cosine

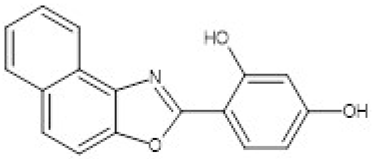

File Name	:	E:\Inne\LR_Lubl\an4461.ms2
File Type	:	Lo-Res Data - Ctd (Magnet)
File Source	:	Acquired on MASPEC II system [II32/99D9]
File Title	:	9 EI 70 eV 33-800
Operator	:	Małgorzata
Instrument	:	AMD 604

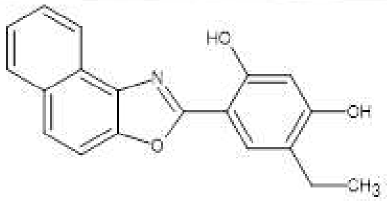

SCAN GRAPH. Flagging=Nominal M/z.

Scan 43#6:19. Entries=890. Base M/z=63.9. 100% Int.=5.824. Temp =261.

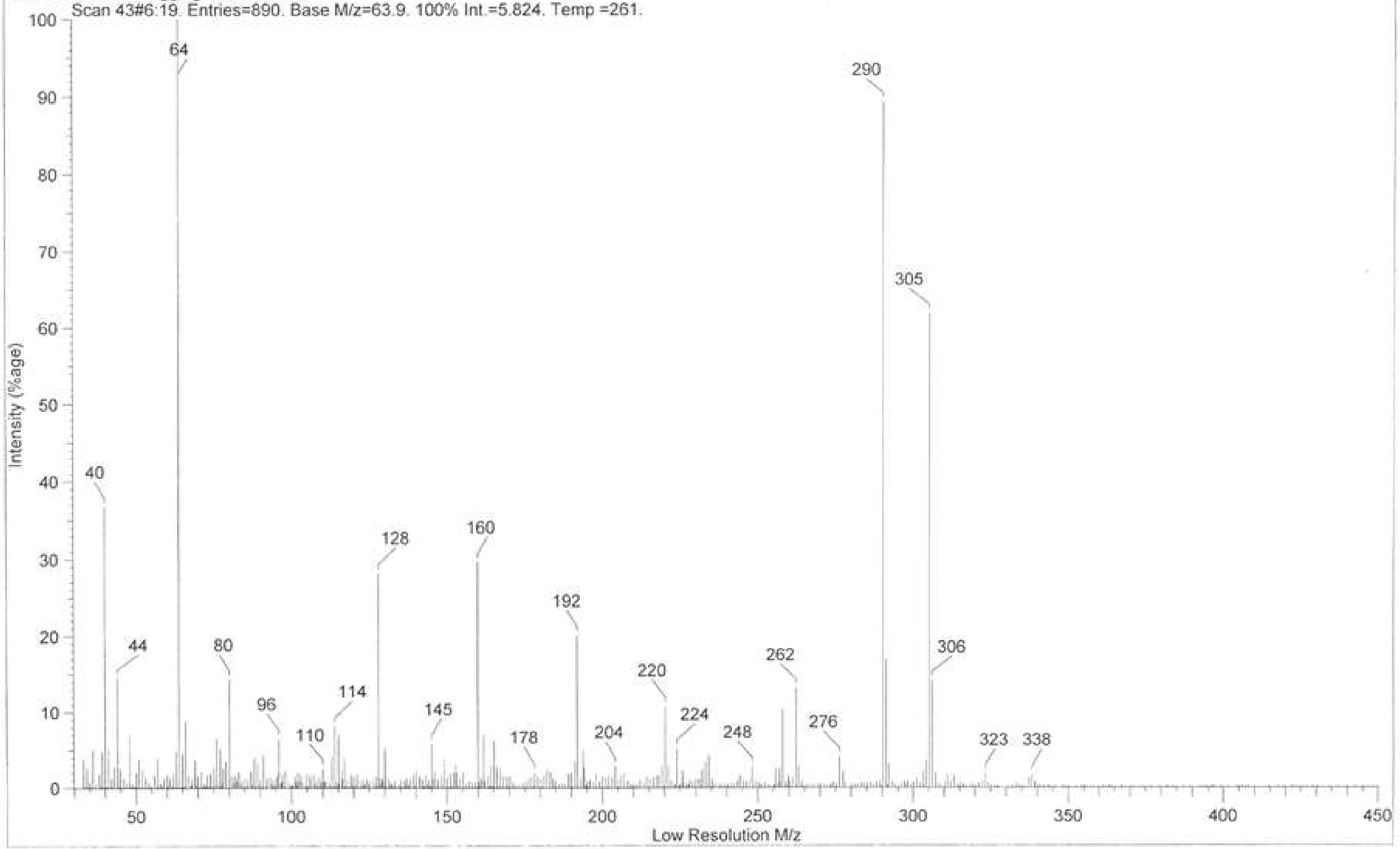

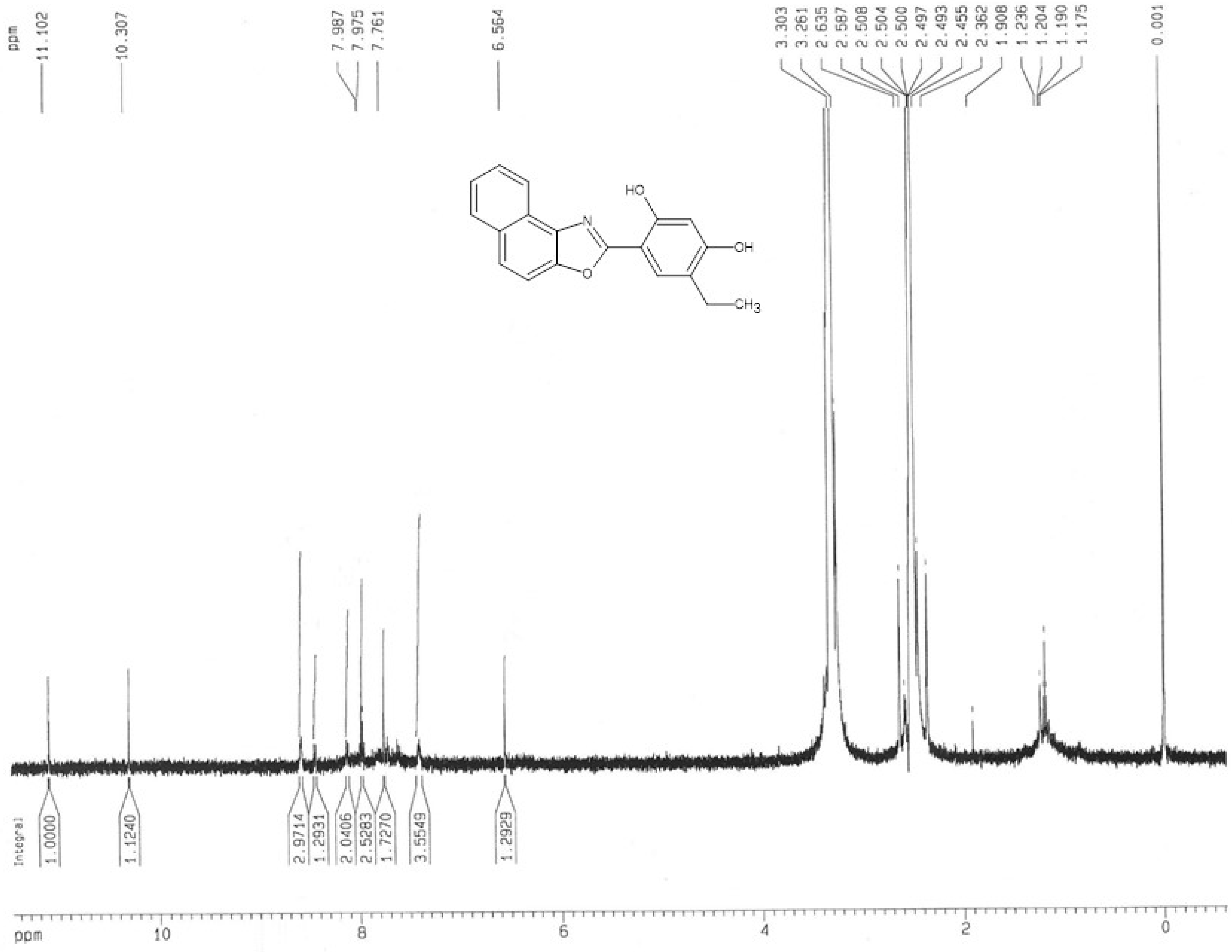

Current Data Parameters

EXPNO	1
PROCNO	1

F2 - Acquisition Parameters

Time	19.22
INSTRUM	spect
PROBHD	5 mm TBI 1H/1
PULPROG	zg
TD	49152
SOLVENT	CDCl3
NS	32
DS	0
SWH	8992.806 Hz
FIDRES	0.182959 Hz
AQ	2.7329011 sec
RG	256
DW	55.600 usec
DE	6.78 usec
TE	303.0 K
D1	1.00000000 sec

============ CHANNEL f1 ============

NUC1	1H
P1	3.00 usec
PL1	3.00 dB
SFO1	500.1340010 MHz

F2 - Processing parameters

SI	65536
SF	500.1300051 MHz
WDW	no
SSB	0
LB	0.00 Hz
GB	0
PC	2.00

1D NMR plot parameters

CX	22.00 cm
F1P	11.478 ppm
F1	5740.57 Hz
F2P	-0.623 ppm
F2	-311.59 Hz
PPMCM	0.55005 ppm/cm
HZCM	275.09811 Hz/cm

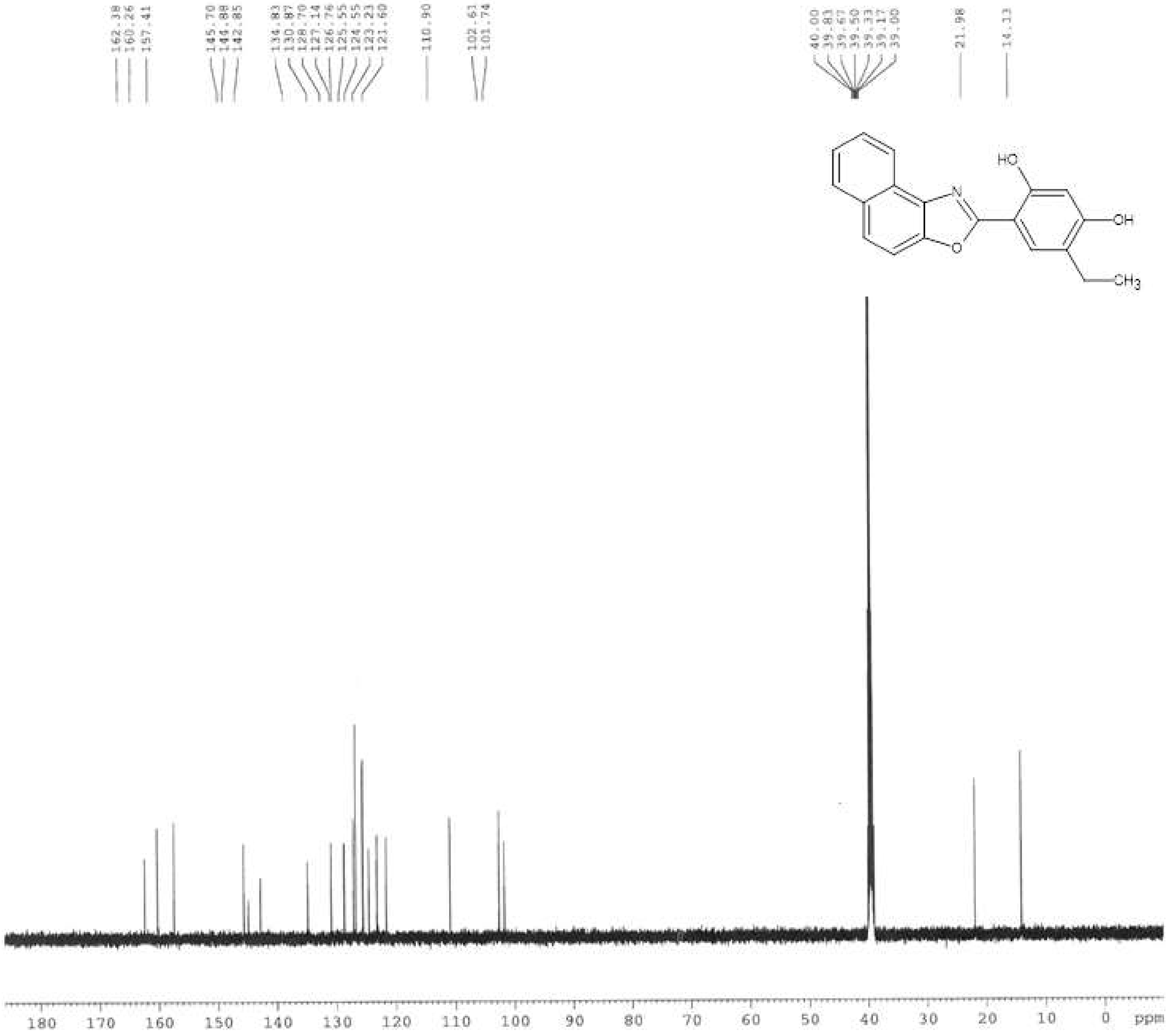

BRUKER

Current Data Parameters

EXPNO	2
PROCNO	1

F2 - Acquisition Parameters

Time	11.58
INSTRUM	DRX
PROBHD	5 mm TBI 1H/13
PULPROG	zgpg
TD	65536
SOLVENT	CDC13
NS	777
DS	4
SWH	32679.738 Hz
FIDRES	0.498653 Hz
AQ	1.0027508 sec
RG	32768
DW	15.300 usec
DE	7.10 usec
TE	303.0 K
D1	1.00000000 sec
d11	0.03000000 sec
DELTA	0.89999998 sec
TD0	1

======== CHANNEL f1 ========

NUC1	13C
P1	5.00 usec
PL1	-3.00 dB
SFO1	125.7703643 MHz

======== CHANNEL f2 ========

CPDPRG2	waltz16
NUC2	1H
PCPD2	98.00 usec
PL2	3.00 dB
PL12	23.00 dB
PL13	32.00 dB
SFO2	500.1320005 MHz

F1 - Acquisition parameters

ND0	1
TD	128
SFO1	500.132 MHz
FIDRES	7.812500 Hz
SW	1.999 ppm
FnMODE	QF

F2 - Processing parameters

SI	262144
SF	125.7578550 MHz
WDW	EM
SSB	0
LB	0.50 Hz
GB	0
PC	1.40

F1 - Processing parameters

SI	1024
MC2	QF
SF	500.1300000 MHz
WDW	SINE
SSB	0
LB	0.30 Hz
GB	0.1

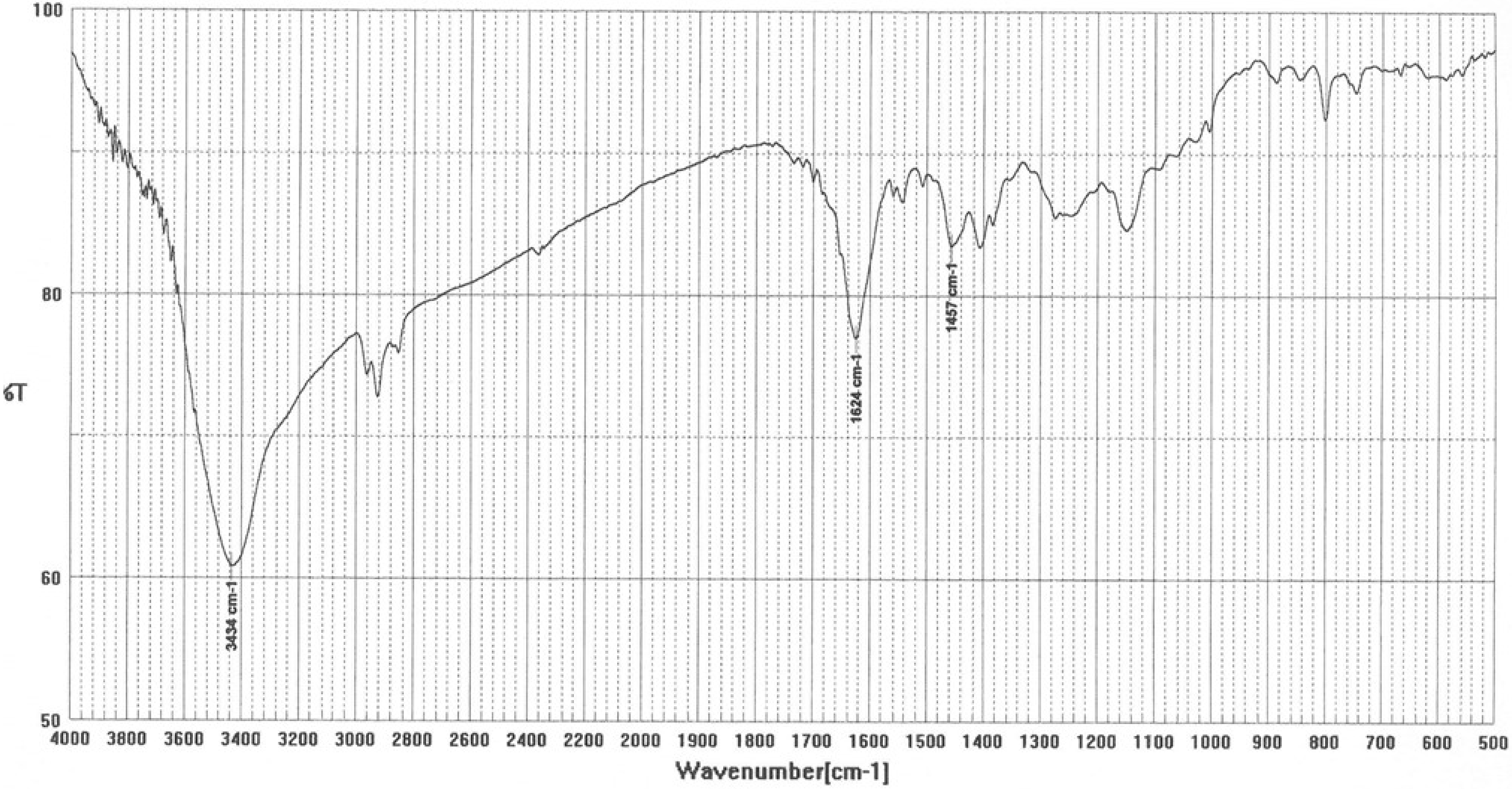

Sample Name	(0,7mg/270mg KBr)
Resolution	1 cm-1
Accumulation	30
Apodization	Cosine

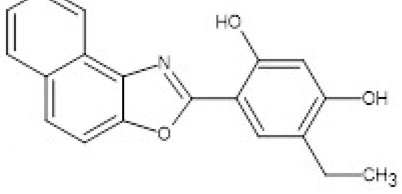

File Name	:	E:\Inne\LR_Lubl\an4460.ms2
File Type	:	Lo-Res Data - Ctd (Magnet)
File Source	:	Acquired on MASPEC II system [II32/99D9]
File Title	:	10 (EI 70 eV 33-800)
Operator	:	Małgorzata
Instrument	:	AMD 604

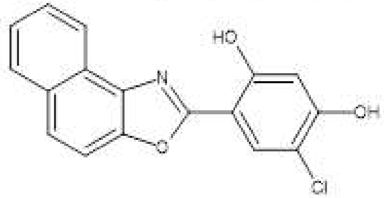

SCAN GRAPH. Flagging=Nominal M/z.

Scan 24#3:31. Entries=774. Base M/z=311.2. 100% Int.=89.6256. Temp =220.

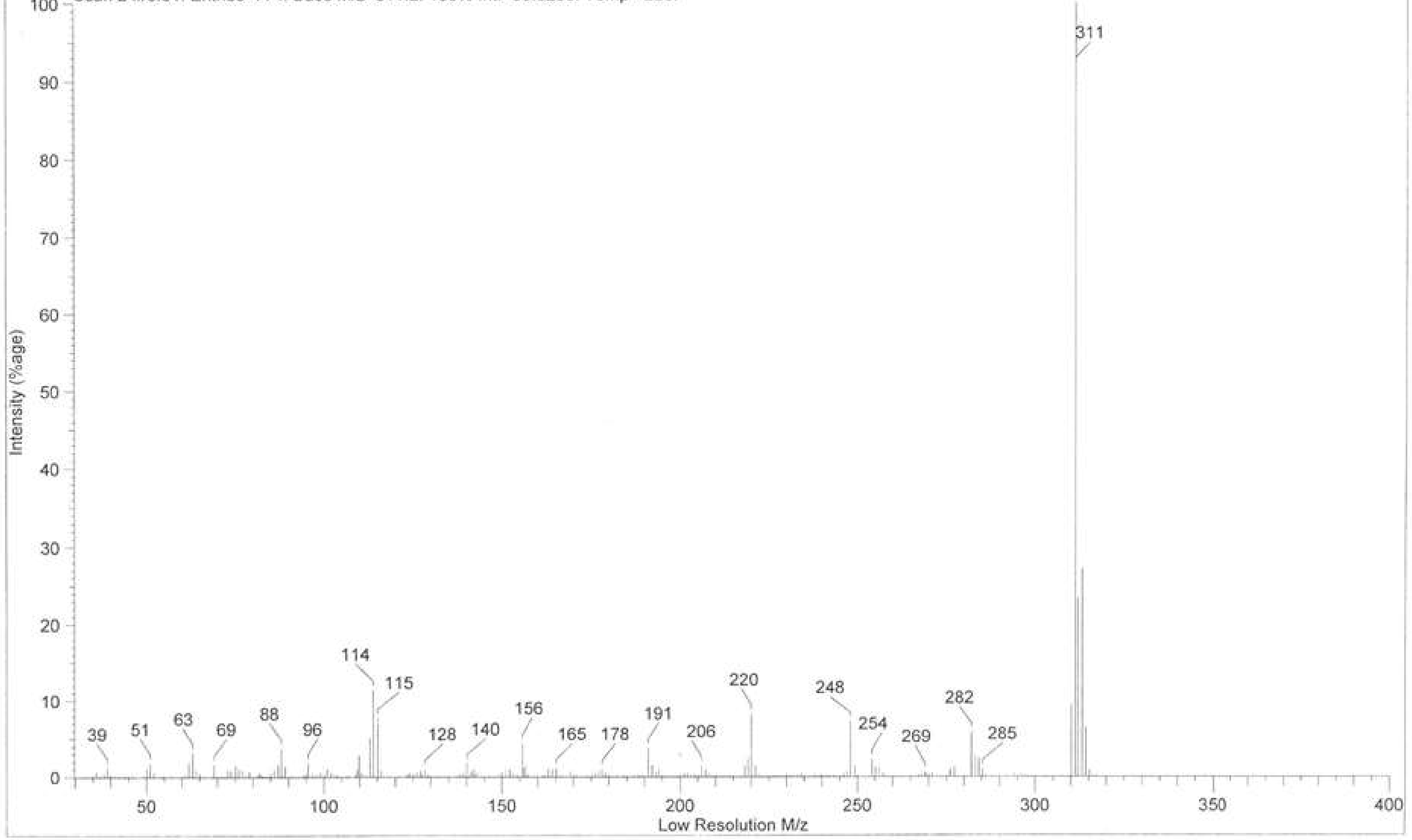

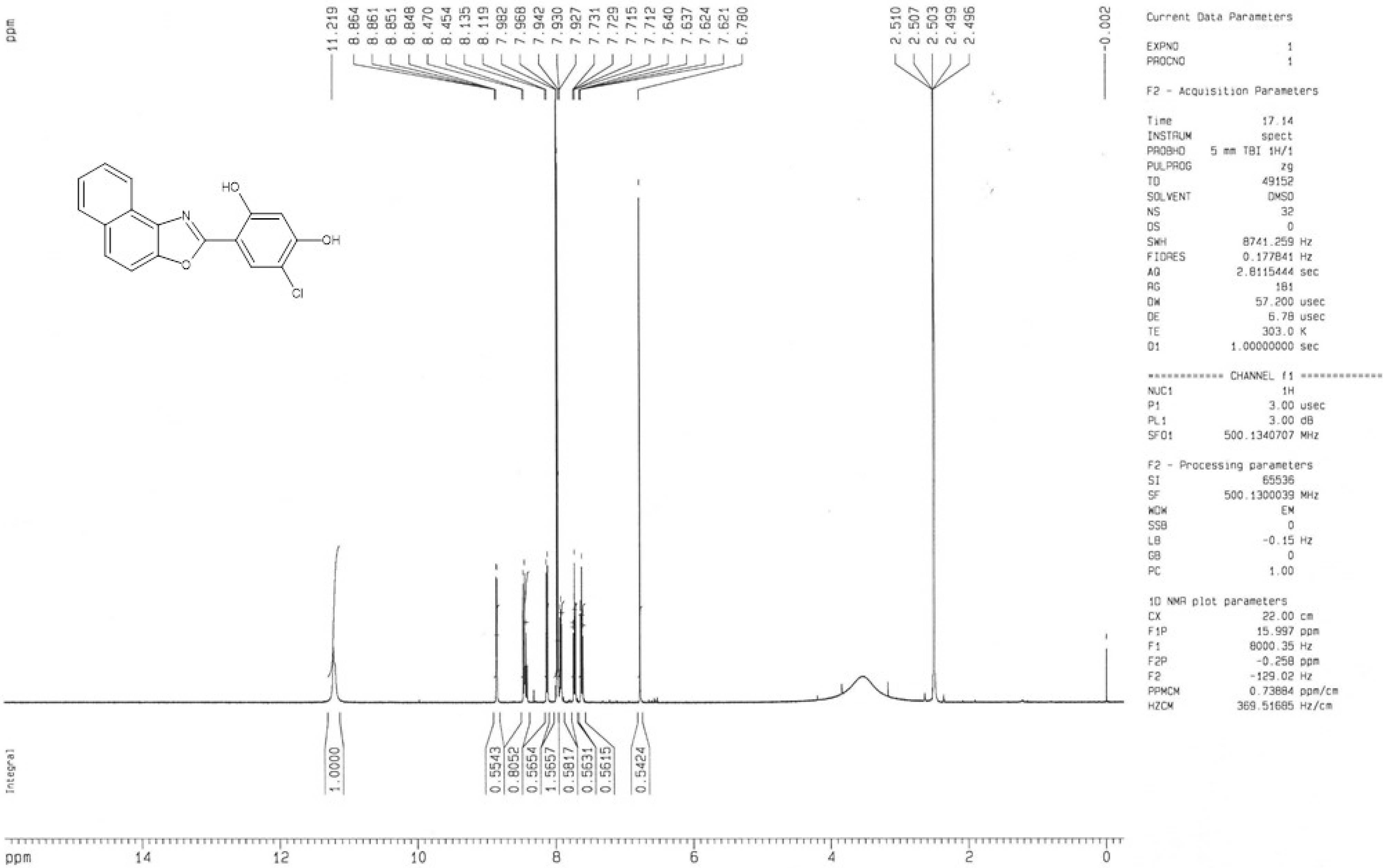

ppm
11.219
8.864
8.861
8.851
8.848
8.470
8.454
8.135
8.119
7.982
7.968
7.942
7.930
7.927
7.731
7.729
7.715
7.712
7.640
7.637
7.624
7.621
6.780
2.510
2.507
2.503
2.499
2.496
-0.002
HO
N
OH
O
Cl
Integral
1.0000
0.5543
0.8052
0.5654
1.5657
0.5817
0.5631
0.5615
0.5424
ppm 14 12 10 8 6 4 2 0
Current Data Parameters
EXPNO 1
PROCNO 1
F2 - Acquisition Parameters
Time 17.14
INSTRUM spect
PROBHD 5 mm TBI 1H/1
PULPROG zg
TD 49152
SOLVENT DMSO
NS 32
DS 0
SWH 8741.259 Hz
FIDRES 0.177841 Hz
AQ 2.8115444 sec
RG 181
DW 57.200 usec
DE 6.78 usec
TE 303.0 K
D1 1.00000000 sec
============ CHANNEL f1 ============
NUC1 1H
P1 3.00 usec
PL1 3.00 dB
SFO1 500.1340707 MHz
F2 - Processing parameters
SI 65536
SF 500.1300039 MHz
WDW EM
SSB 0
LB -0.15 Hz
GB 0
PC 1.00
1D NMR plot parameters
CX 22.00 cm
F1P 15.997 ppm
F1 8000.35 Hz
F2P -0.258 ppm
F2 -129.02 Hz
PPMCM 0.73884 ppm/cm
HZCM 369.51685 Hz/cm

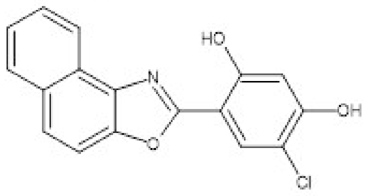

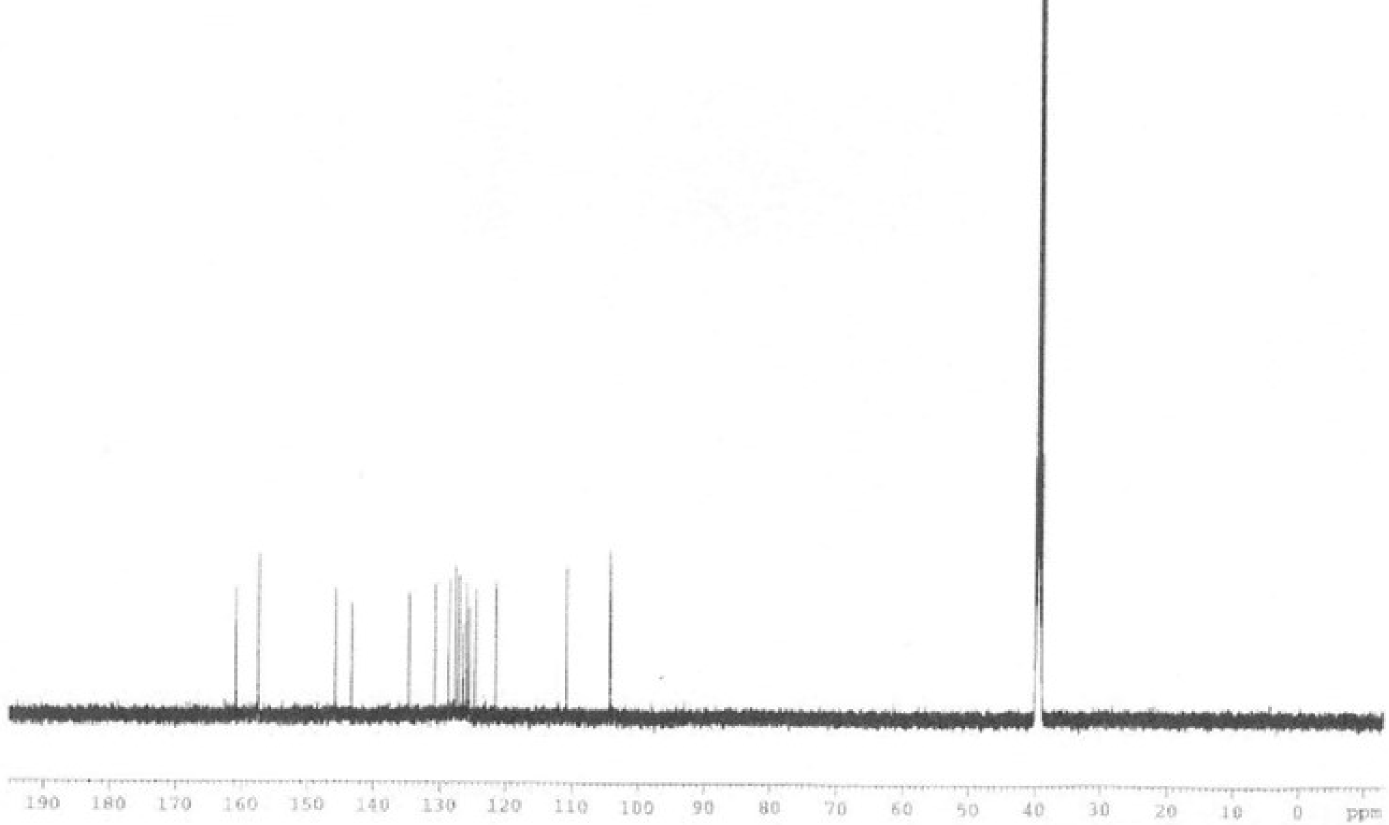

BRUKER

Current Data Parameters

```
EXPNO                  2
PROCNO                 1

F2 - Acquisition Parameters

Time               13.31
INSTRUM              DRX
PROBHD   5 mm TBI 1H/13
PULPROG             zgpg
TD                 65536
SOLVENT            CDCl3
NS                   620
DS                     4
SWH           32679.738 Hz
FIDRES         0.498653 Hz
AQ            1.0027508 sec
RG                 32768
DW                15.300 usec
DE                  7.10 usec
TE                 303.0 K
D1            1.00000000 sec
d11           0.03000000 sec
DELTA         0.89999998 sec
TD0                    1

======== CHANNEL f1 ========
NUC1                 13C
P1                  5.00 usec
PL1                -3.00 dB
SFO1        125.7703643 MHz

======== CHANNEL f2 ========
CPDPRG2          waltz16
NUC2                  1H
PCPD2              98.00 usec
PL2                 3.00 dB
PL12               23.00 dB
PL13               32.00 dB
SFO2        500.1320005 MHz

F1 - Acquisition parameters
ND0                    1
TD                   128
SFO1             500.132 MHz
FIDRES          7.812500 Hz
SW                 1.999 ppm
FnMODE                QF

F2 - Processing parameters
SI                262144
SF          125.7578544 MHz
WDW                   EM
SSB                    0
LB                  0.50 Hz
GB                     0
PC                  1.40

F1 - Processing parameters
SI                  1024
MC2                   QF
SF           500.1300000 MHz
WDW                 SINE
SSB                    0
LB                  0.30 Hz
GB                   0.1
```

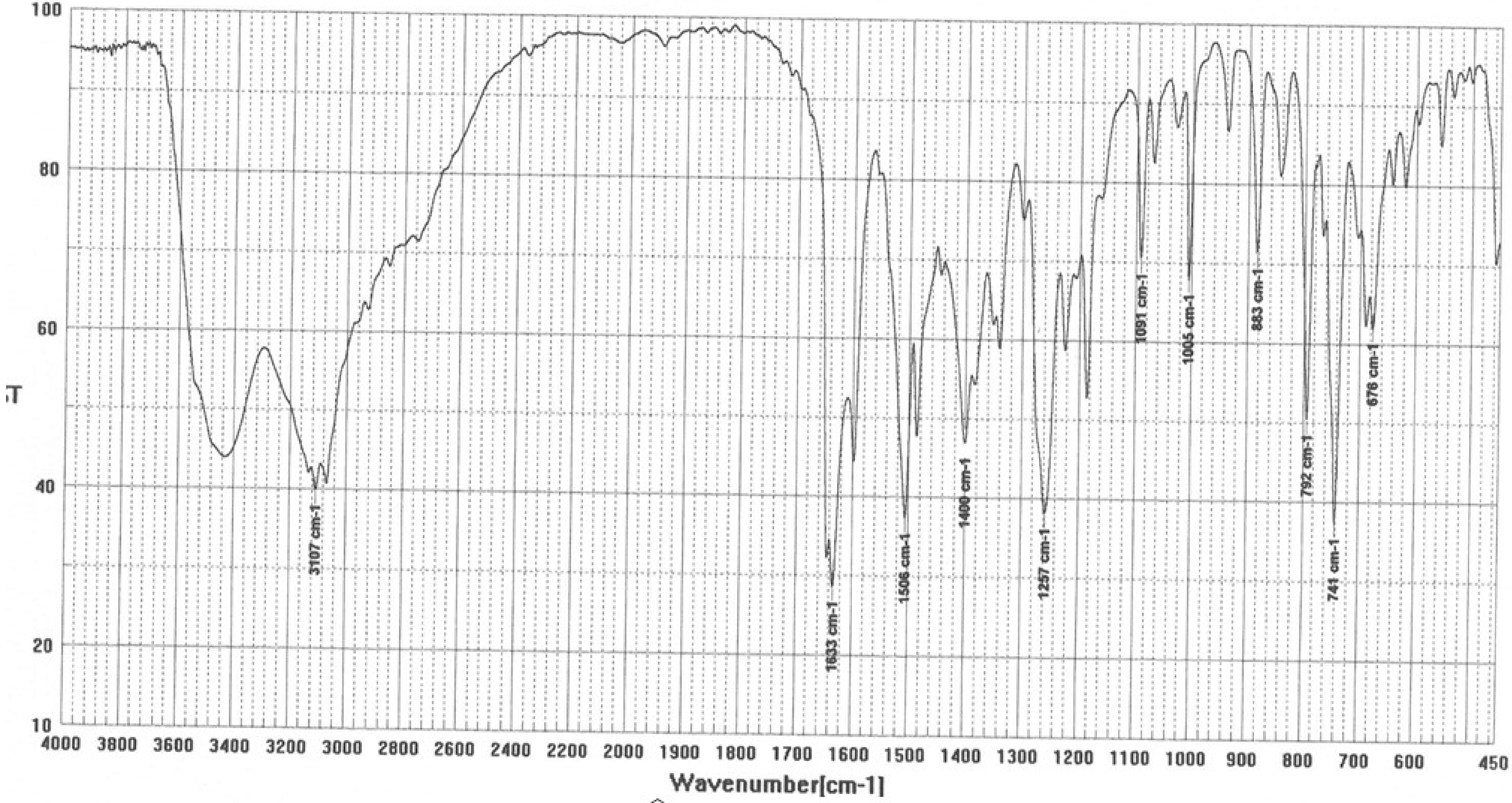

Sample Name	0,8mg/270mgKBr	
Resolution	1 cm-1	
Accumulation	30	
Apodization	Cosine	

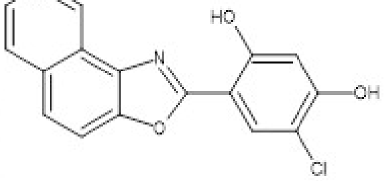

Supplement: Supplementary file 1 [file molecules-27-08511-s001.zip › molecules-2036572-supplementary materials.pdf]
